# Modelling *Salmo trutta* Complex Spatial Distribution in Central Italy: A Random Forest Approach Revealing Underrepresented Lowland Populations Based on Spatially‐Explicit Predictors

**DOI:** 10.1002/ece3.71658

**Published:** 2025-06-26

**Authors:** Lorenzo Talarico, Elena Catucci, Marco Martinoli, Michele Scardi, Lorenzo Tancioni

**Affiliations:** ^1^ Department of Biology University of Rome ‘Tor Vergata’ Rome Italy; ^2^ National Inter‐University Consortium for Marine Sciences (CoNISMa) Rome Italy; ^3^ Council for Agricultural Research and Economics Research Centre for Animal Production and Aquaculture Monterotondo Italy

**Keywords:** bioclimatic constraints, brown trout, habitat suitability, machine learning, species distribution modelling

## Abstract

Species distribution models are powerful tools to infer ecology and support management of conservation and socio‐economic valuable taxa, such as brown trout ( *Salmo trutta* complex). Using a random forest approach, we modelled its distribution in central Italy watercourses, using recent presences/absences and eight environmental/bioclimatic predictors. The model shows (i) high predictive ability (*K* = 0.76), (ii) predicts suitable, naturally‐infrequent lowland watercourses where brown trout occurs or may occur. Moreover, the prediction values (iii) expresses a remarkable positive monotone relationship with abundance classes of brown trout computed during field sampling, despite such information was not included in the model development. Predictors' importance pointed out to the crucial role of bioclimatic constraints (linked to thermal suitability and habitat availability) over anthropogenic disturbance and lithotypes. This modelling exercise reiterates the importance of modelling approaches based on spatially explicit proxies of species habitat requirements to assist taxa management by revealing suitable but infrequent and singular areas that could be considered worthy of protection.

## Graphical abstract

Using freely available spatially explicit environmental‐bioclimatic predictors, in a Random Forest framework, we: (i) accurately modelled the spatial distribution of *Salmo trutta* complex in central Italy; (ii) revealed the suitability of naturally infrequent lowland watercourses where trout occurrence is only partly known; (iii) provided information on relative contributions of predictors; (iv) detected a relationship between model predictions and brown trout abundance classes.

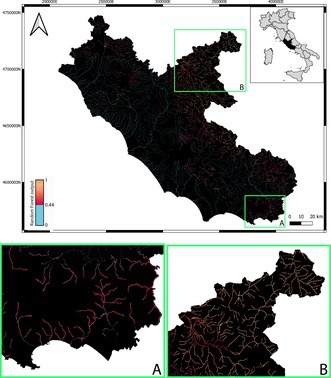

## 1 Introduction

In the last decades, species distribution models (SDMs) have become valuable tools for enhancing biodiversity management and conservation, with special emphasis on priorities such as endangered, invasive or overexploited taxa (Rodríguez et al. 2007; Dawson et al. 2011). Based on occurrence data and through various methodological approaches (e.g., linear vs. machine learning), SDMs may allow the identification of suitability areas for the presence of a given taxon, while contextually determining the most informative environmental, bioclimatic or anthropic drivers to describe its habitat requirements (e.g., Mouton et al. 2011; Filipe et al. 2013; Muñoz‐Mas et al. 2016). From a conservation perspective, such information may ultimately contribute to the identification of pivotal areas for species protection, as well as promising sites for future sampling (Rodríguez et al. 2007).

Brown trout ( *Salmo trutta* species complex) is an intensively managed Salmonid, introduced and naturalised almost worldwide (expanding its native range that roughly covered the Western Palearctic region; MacCrimmon and Marshall 1968) because of its ecological plasticity and considerable socio‐economic value as food and recreational (angling) resource. On the other hand, multiple taxa of the species complex have received conservation attention due to their ecological and/or genetic distinctiveness, often associated with limited distribution (Lobón‐Cerviá 2018). A multitude of studies investigated brown trout ecology at various spatio‐temporal scales and life stages, revealing the important role of abiotic features—among which water temperature, flow, depth, bottom substrate, watercourse's width and slope—and (palaeo)climate conditions—such as air temperature, precipitations and ice cover—to determine distribution and abundance of wild populations (e.g., MacCrimmon and Marshall 1968; Mäki‐Petäys et al. 1997; Vismara et al. 2001; Mouton et al. 2011; Filipe et al. 2013; Cianfrani et al. 2015; Muñoz‐Mas et al. 2016; Splendiani et al. 2016). Importantly, studies demonstrated fascinating variation in life histories and habitat preferences among locations, likely as a consequence of site‐specific environmental characteristics and opportunistic fish behaviour (reviewed in Jonsson and Jonsson 2018).

In Italy, the native brown trout—referred to as *S. ghigii* and listed as critically endangered by the Italian IUCN committee (Rondinini et al. 2022), although taxonomy remains controversial—mainly inhabits mountain watercourses or naturally less‐frequent watercourses originating from karstic springs at medium‐low altitudes (Zanetti 2016 and citations therein) and even close to the coasts (e.g., Fabiani et al. 2018; Rossi et al. 2019, 2022). Italian peninsular brown trout populations generally occur in cold (optimal range is approximately 10°C–17°C), transparent and well‐oxygenated flowing waters (Zanetti 2016; Lorenzoni et al. 2019), even if Sardinian trout are known to reside in stagnant pools during summer droughts, tolerating up to 21°C–23°C water temperature and < 6 mg/L dissolved oxygen (Zaccara et al. 2015 and references therein). Brown trout are resident or potamodromous in the Mediterranean area, and so are in Italy, meaning they complete their life cycle entirely in freshwater, possibly migrating (usually for short distances) towards small tributaries for breeding during winter in unfragmented watercourses (Kottelat and Freyhof 2007; Aparicio et al. 2018). However, life history traits and actual habitat preferences of native Italian brown trout remain poorly known, and their understanding may be hindered by the confounding effect of stocking activities (Lorenzoni et al. 2019), as the systematic introduction of hatchery trout of Atlantic origin ( *S. trutta* sensu stricto) since the 19th century and up to approximately 2020 has resulted in naturalised populations and widespread introgression in wild populations (Splendiani et al. 2016; Polgar et al. 2022).

Here, by using public information on species occurrence (i.e., presence/absence data) from field surveys over the last decades, we developed a random forest‐based framework to predict the spatial distribution of *S. trutta* complex (brown trout, hereafter) in central Italy (Latium Region), where populations occur in both mountain‐typical and lowland naturally‐infrequent areas. Although species origin often remains unassessed within our study area, to date, genetic studies have revealed a few native‐residual populations at lower altitudes, and widespread introgressed populations previously subject to stocking with Atlantic hatchery trout (Fabiani et al. 2018; Rossi et al. 2019, 2022)—conversely, less introgressed populations were found at higher altitudes of remote watercourses from the central Adriatic slope (Lorenzoni et al. 2019). For modelling development, we used bioclimatic/environmental variables along with human footprint and lithotype, and assessed their relative importance in determining the suitability of habitat conditions. Outcomes from this study would help define major drivers of brown trout's distribution while identifying suitable unexplored areas where populations may occur, particularly in infrequent but suitable lowlands or unprotected areas.

## 2 Materials and Methods

### 2.1 Brown Trout Distribution Data

**FIGURE 1:**
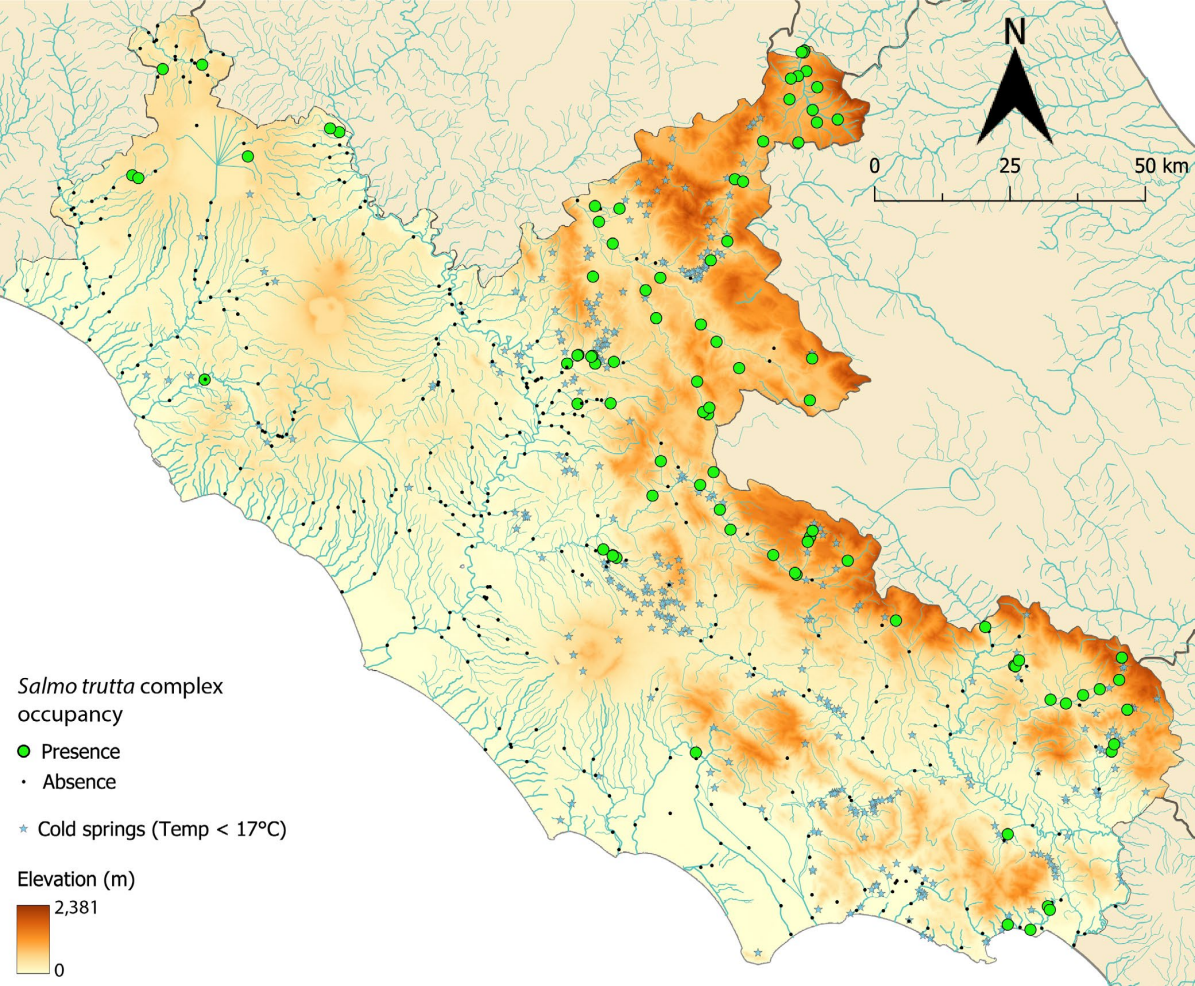
Spatial distribution of 406 sampling sites across Latium watercourses in central Italy with information on elevation. The location of cold springs (gushing water temperature < 17°C) is shown as well–data from https://geoportale.regione.lazio.it/layers/geonode:Carta_idrogeologica_sorgenti_puntuali).

We took advantage of public data on brown trout distribution in Latium (central Italy) obtained from electrofishing‐based fish surveys performed approximately over the last 20 years to assess the composition and abundance of fish communities across regional lotic freshwaters (such as creeks, streams, rivers, drains, etc.—https://geoportale.regione.lazio.it/layers/geonode:Biodiversit_ittica_acque_correnti#more), partly in compliance with the Habitat Directive (92/43/EEC). We also included 11 recent presences (Tancioni & Talarico, unpublished data). Overall, presences accounted for ~22% of the total sampling sites, namely 88 records over 406 (Figure 1).

Worth noting that presence data do not distinguish among Mediterranean‐native (*S. ghigii*), entirely stocked (i.e., with Atlantic‐exotic *S. trutta* , sensu stricto) or admixed populations, as genetic‐diagnostic information is restricted to a few investigated sites (e.g., Fabiani et al. 2018; Rossi et al. 2019, 2022). Hence, the majority of surveys relied on morphological inspection of specimens only, without providing information of their genetic. Analogously, we could not verify the viability of wild populations because the available information on demographic structure (e.g., frequency/distribution of length‐age classes, occurrence of juveniles) is highly fragmentary and currently not digitalised (e.g., Sarrocco et al. 2012).

### 2.2 Predictive Variables

To ensure the generality of our model and its applicability to unsampled and/or unexplored areas, we only used public, spatially explicit predictors with global coverage and a wide temporal scale. Specifically, we considered seven bioclimatic/environmental variables (Table 1) which are known to affect species presence (e.g., MacCrimmon and Marshall 1968; Splendiani et al. 2013, 2016). As brown trout is a cold‐water taxon, we primarily included the maximum temperature of the warmest month and the minimum temperature of the coldest month to account for possible critical ranges of water temperatures, as air temperature was found to adequately approximate inland water temperature (e.g., Cianfrani et al. 2015). Secondly, we included the elevation that, besides an obvious correlation with air temperature (Figure S1), intrinsically expresses various hydromorphological features (such as slope, river width, flow and bottom substrate) according to longitudinal river zonation (Illies and Botosaneanu 1963). The annual precipitation was assumed as a proxy for water flow (Dettinger and Diaz 2000; Filipe et al. 2013), while precipitations of the driest and wettest months were included to account for limiting water flow fluctuations (i.e., droughts and floods, respectively). We also considered ‘simplified’ lithology (six lithotypes: carbonate, allochthonous flysch, arenaceous flysch, clay flysch, post‐orogenic units, volcanic), which is linked to water parameters such as pH and organic dissolved matter (Thomas et al. 1999; Mosher et al. 2010) and appeared related to the occurrence of Italian‐native brown trout (Splendiani et al. 2013, 2016). Finally, to account for anthropogenic disturbance, we included the human footprint (Venter et al. 2016) that combines information from eight human pressures (i.e., built environments, population density, electric infrastructure, crop lands, pasture lands, roads, railways and navigable waterways).

**TABLE 1:** Details on variables used to predict suitable habitat for brown trout, along with their relative importance according to Gini and Permutation measures (top‐four predictors for the two measures are given in bold).

Variable—description	Period	Unit	Native resolution	Source	Link	Importance % (Gini)	Importance % (Permutation)
**BIO5**—Maximum temperature of the warmest month	1970–2000	°C	1 km	WorldClim 2.1	https://www.worldclim.org/data/worldclim21.html#google_vignette	**18.2**	**15.2**
**BIO6**—Minimum temperature of the coldest month	1970–2000	°C	1 km	WorldClim 2.1	https://www.worldclim.org/data/worldclim21.html#google_vignette	**26.9**	**18.6**
**BIO12**—Annual precipitation	1970–2000	Millimetres	1 km	WorldClim 2.1	https://www.worldclim.org/data/worldclim21.html#google_vignette	8.9	11.8
**BIO13**—Precipitation of the wettest month	1970–2000	Millimetres	1 km	WorldClim 2.1	https://www.worldclim.org/data/worldclim21.html#google_vignette	8.0	10.5
**BIO14**—Precipitation of the driest month	1970–2000	Millimetres	1 km	WorldClim 2.1	https://www.worldclim.org/data/worldclim21.html#google_vignette	**11.9**	**20.0**
**DEM** (Tinitaly/1.1)—Elevation	—	Metres	10 m	Tarquini et al. (2023)	https://tinitaly.pi.ingv.it/Download_Area1_1.html	**17.5**	**14.4**
**Lithotype**	1990	Nominal	Vectorial	Geoportale Regione Lazio	https://geoportale.regione.lazio.it/layers/geonode:cig_basi_geologiche/metadata_detail	1.8	3.3
**HF2009**—Human footprint	2009	HF index	1 km	Venter et al. (2016)	https://datadryad.org/stash/dataset/doi:10.5061/dryad.052q5	6.8	6.2

Essential information on predictive variables—such as source, description and spatial resolution—is shown in Table 1 and Figure S1, while the complete input dataset is provided in Table S1. The predictive variables were interpolated to obtain a final resolution of 100 m, regardless of their native spatial resolution—for the elevation only, we performed a resample (from 10 m to 100 m resolution) to maintain spatial consistency among predictors.

### 2.3 Model Development and Validation

Although available data on brown trout distribution included semi‐quantitative abundances (i.e., abundance classes), we developed the model using binary (presence/absence) data only. As a matter of fact, information on brown trout abundance from field sampling was exploited to detect a potential relationship with model prediction values.

To model the spatial distribution of such species, we adopted a Random Forest (RF) classification approach (Breiman 2001), a machine learning algorithm that is widely used in SDM.

To improve the accuracy of the RF model, we tuned the number of randomly selected predictors (*mtry*) and the minimum number of cases to stop the splitting (*nodesize*). We tested the *mtry* in the [3,6] range of values, while the *nodesize* in the [1,10] range, setting the total number of trees in the forest (*ntree*) to 500.

To evaluate the contribution of predictors we used: (i) the Gini measure which assesses the relative importance of a predictive variable based on the overall decrease in Gini impurity over the forest (Breiman et al. 1984) scaled by the total number of trees; (ii) the Permutation measure that mimics the absence of a given predictor providing a measurement of its relative importance based on the change in the prediction accuracy (Boulesteix et al. 2012)—for details see Louppe et al. (2013) and references therein.

We partitioned the data into the training subset (~75%) for model development and the test subset (~25%) for model validation, as follows. The study area was split into 63 grid cells of size 20 × 20 km (Figure S2). To account for the biased spatial distribution of the presences—concentrated in hilly‐mountain areas of the northeastern part of the Latium Region (Figure 1)—we divided the data into four geographic quadrants (e.g., Osborne and Suárez‐Seoane 2002) (Figure S2) obtaining: 51 records (presences = 78.4%) across 12 cells within the North‐East quadrant; 119 records (presence = 24.4%) across 21 cells in the South‐East quadrant; 48 records (presence = 8.3%) across 9 cells in the South‐West quadrant; 188 records (presence = 8.0%) across 21 cells in the North‐West quadrant. Finally, we randomly assigned about 25% of records to the test set within each quadrant, maintaining the relative presence/absence ratio.

This procedure allowed avoiding as much as possible the effects of spatial autocorrelation and preserving the representativeness of input data (i.e., the spatial distribution of brown trout) during both RF training and validation procedures.

Before evaluating model accuracy, the prediction values (i.e., RF output, hereafter) was optimised using the Receiver Operating Characteristic (ROC) curve, which allows to properly deal with an imbalanced data set (e.g., Catucci and Scardi 2022). In fact, the ROC curve provides the value of the optimal threshold for distinguishing between presence and absence records, that is the point for which the sum of sensitivity and specificity is maximised. Once the optimal threshold was selected, the accuracy of the RF models was assessed by the *K* metric, computed using the test dataset only (Cohen 1960). The best model was the one showing the highest predictive ability, namely the largest *K*.

Model development and validation were performed with the ‘*randomForest*’ package (Liaw and Wiener 2002) in R (R Development Core Team 2022).

## 3 Results

**FIGURE 2:**
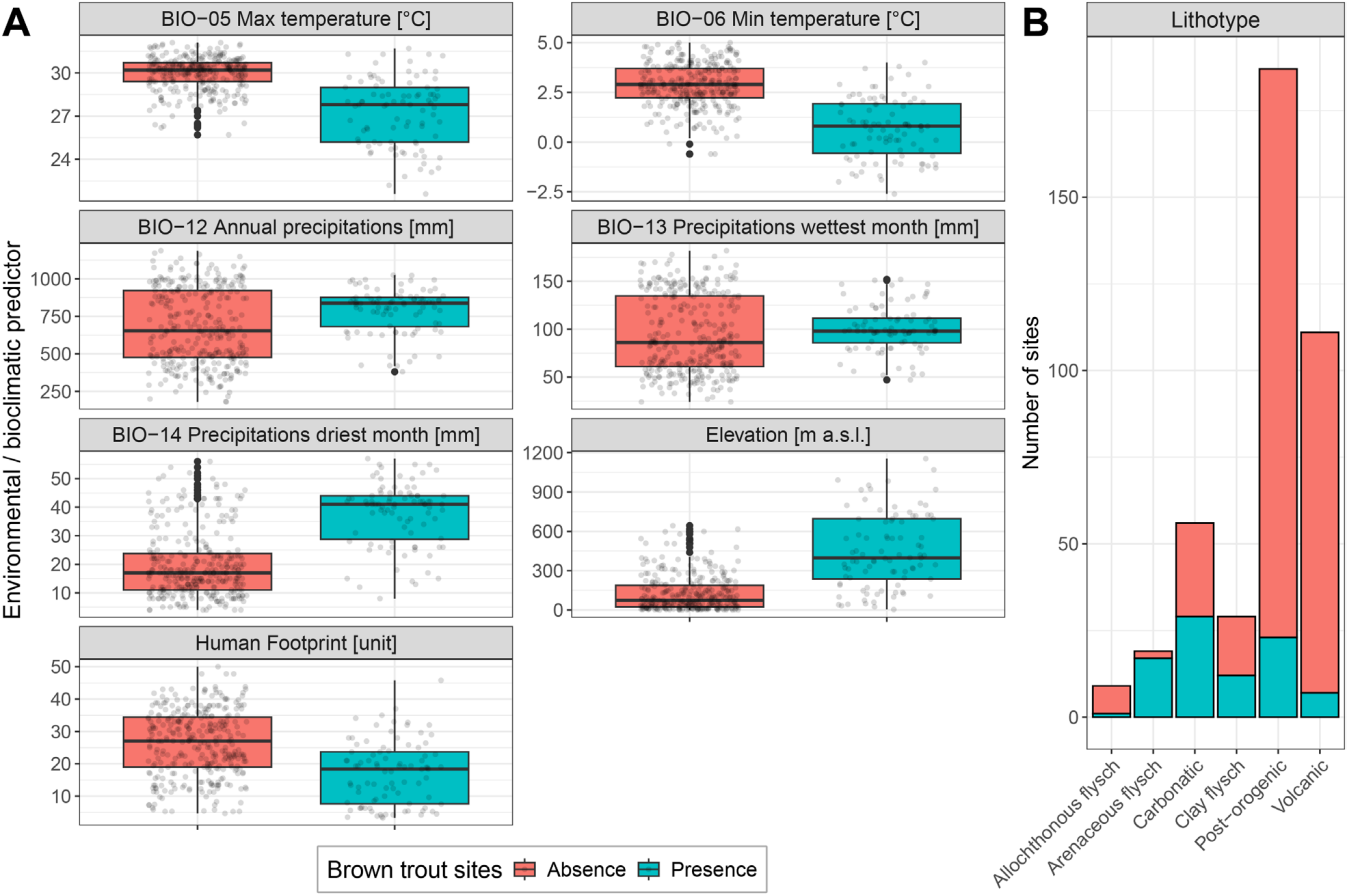
Distribution of *S. trutta* complex presences (*N* = 88) and absences (*N* = 318) for each of eight predictors: (A) boxplots of six environmental/bioclimatic variables and the anthropogenic disturbance compound index; (B) bar charts of lithological categories.

A preliminary visual inspection revealed different distributions of brown trout presences as opposed to absences for accounted predictors, except BIO12 and BIO14 (Figure 2).

**FIGURE 3:**
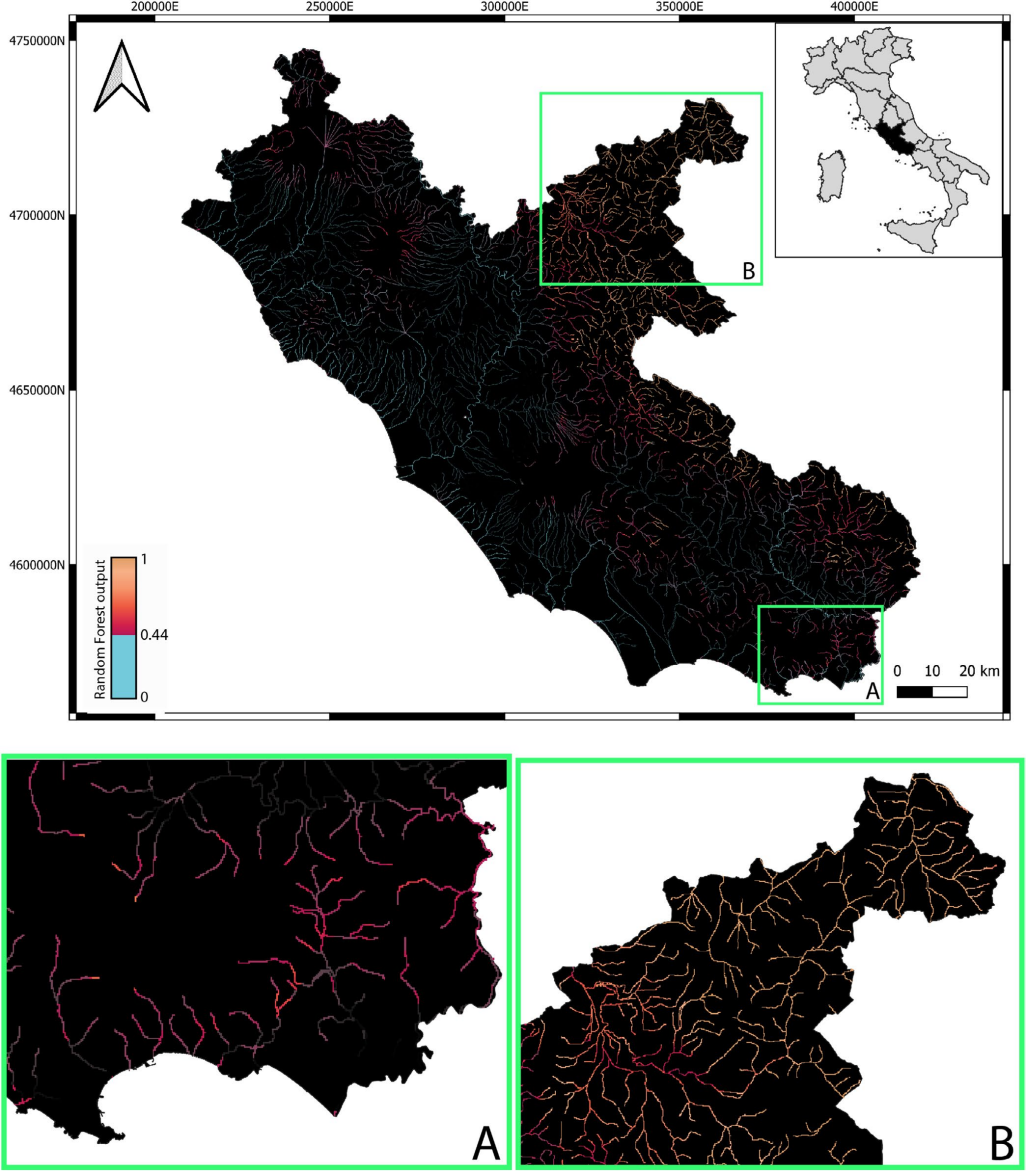
Predicted spatial distribution of *S. trutta* complex across Latium watercourses—for better visualisation, unsuitable areas (RF output < 0.44) are indicated in light blue. Enlarged maps of the southern coast (A) and central Apennine (B) areas are shown below.

The ROC curve (Figure S3) analysis, performed to account for unbalanced occurrences, returned an optimal threshold of 0.44 (AUC = 0.94) for discriminating between presence and absence instances. The final model showed large values of accuracy in terms of sensitivity (0.87), specificity (0.92) and mostly *K* (0.76), pointing out high predictive ability. The final RF configuration is based on 500 trees with both *mtry* and *nodesize* equal to 4. The threshold optimisation selection allowed for an approximately 10% reduction of the false rate (Table S2), thereby improving the model's sensitivity and capacity to detect suitable habitats. The RF model predicted about 24% as suitable areas for brown trout within Latium watercourses (Figure 3). As expected, the model found mountain creeks and streams as generally highly suitable (e.g., Figure 3B), and interestingly it also revealed non‐typical lowland and coastal areas (e.g., Figure 3A).

The relative importance of predictive variables for Gini and Permutation measures is given in Table 1. The four most important variables, the same for the two methods, cumulatively accounted for ~74% and ~68% of total importance. The minimum temperature of the coldest month (BIO6, ~27%) and precipitation of the driest month (BIO14, ~20%) ranked as the most important according to Gini and Permutation, respectively. Conversely, the importance of lithotypes (1.8% and 3.3%) and human footprint (6.8% and 6.2%) was negligible for both measures.

## 4 Discussion

We developed a random forest‐based model to predict the distribution of brown trout ( *S. trutta* in central Italy (Latium Region) while accounting for spatially unbalanced occurrences of input data. To do so, we used freely available, spatially‐explicit predictive variables with robust ecological soundness for brown trout and evaluated their relative importance.

**FIGURE 4:**
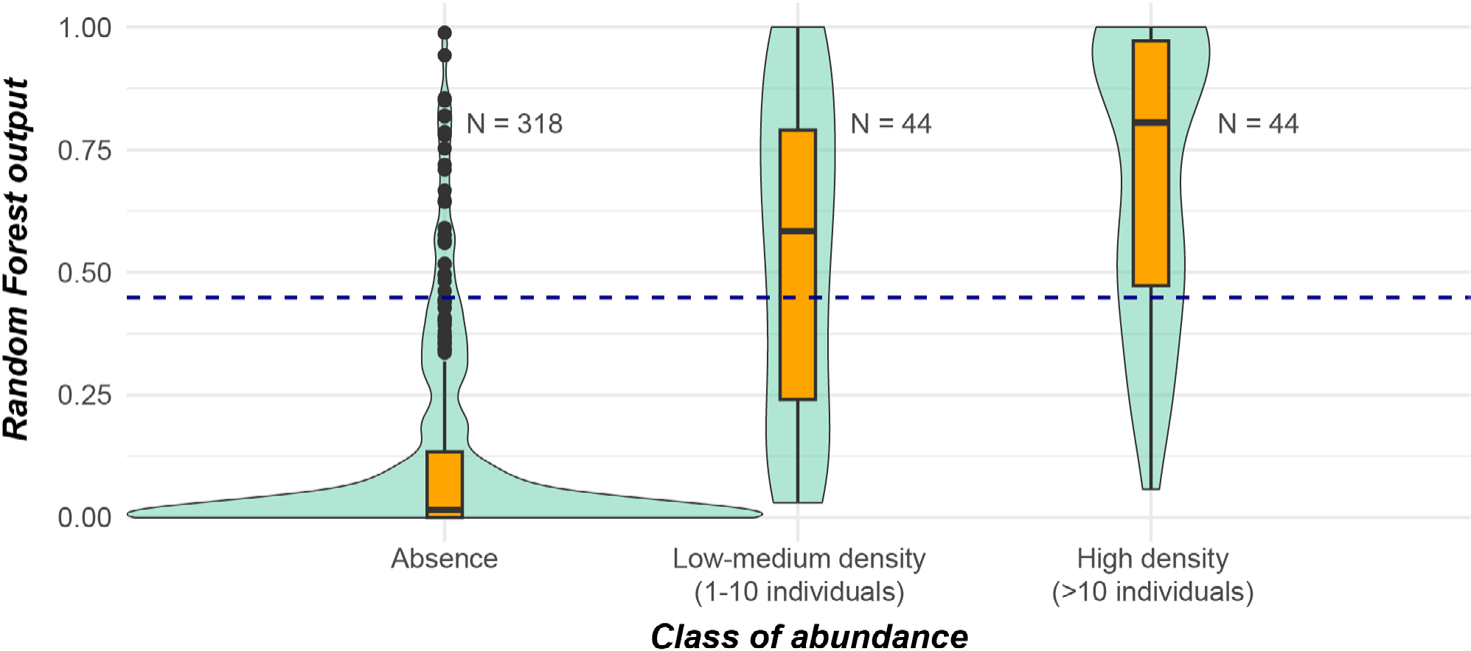
Box and violin plots indicate the distribution of RF output at three increasing *S. trutta* complex abundance classes (absence, low‐medium density, high density) derived from field sampling. Medians of distributions were statistically different overall (Kruskal‐Wallis test: *H* = 145.2, *p* < 0.0001) and between pairs (Mann–Whitney tests: 678 < *U* < 1587, *p* < 0.0001 using Bonferroni correction). The sample size for each abundance class and the optimal threshold (horizontal blue dashed line) for RF output are shown.

The high accuracy of the obtained model (*K* = 0.76) revealed its effectiveness. Moreover, a clear positive relationship emerged by analysing the distribution of RF output at increasing abundance classes derived from field sampling (Figure 4)—medians of predictions were statistically different among classes of abundance (Kruskal–Wallis, *p* < 0.0001) and between pairs (Mann–Whitney tests: *p* < 0.0001 using Bonferroni correction). This is remarkable since class abundances were not taken into account in the modelling development, suggesting that the RF output intrinsically expressed information on semi‐quantitative abundances of brown trout (e.g., Catucci and Scardi 2022).

The model predicted, as expected, Apennine areas as highly suitable for brown trout (e.g., Figure 3B). Mountain watercourses are indeed a typical Salmonid habitat (Huet 1959) in Italy, as altitude usually implies cold, well‐oxygenated and unpolluted waters in spring‐rich, relatively undisturbed environments (except for the occurrence of physical and hydraulic anthropic barriers) (Carosi et al. 2020). Besides, the model interestingly revealed some potentially suitable watercourses at low altitudes where brown trout actually occurs (e.g., the southern Latium coastal area, Figure 3A), or occurred historically (e.g., in the Pontina Plain; Vinciguerra 1902), or has not been recorded/investigated to date (e.g., northern Latium coastal and lowland areas). This may be due to the occurrence of lowland cold‐water refugia (Figure 1) or local populations tolerating relatively warm waters—for example, Sardinian trout can reside in standing puddles with reduced oxygen saturation, no flow and relatively high water temperature (20°C–23°C) during summer droughts (Zaccara et al. 2015). Remarkably, analysed lowland and coastal populations of brown trout in Latium showed native‐genetic distinctiveness and little introgression from domestic‐exotic strains, hence deserving special conservation attention (e.g., Capodacqua and Rio Santa Croce populations; Fabiani et al. 2018; Rossi et al. 2019, 2022). This would eventually stimulate further sampling in less‐explored putatively suitable areas—such as central and northern Latium lowland and coastal areas and, by extension, other comparable Italian watercourses—especially outside protected areas.

The relative values of Gini and Permutation measures suggested the prominent role, in terms of relative importance, of bioclimatic and environmental variables over anthropogenic disturbance and lithology.

Extremes of air temperature, that is known to adequately approximate water temperature (Cianfrani et al. 2015), appeared particularly informative to determine brown trout distribution in our model. The minimum temperature of the coldest month (BIO6 contribution = 26.9% for Gini and 18.6% for Permutation), which falls in winter and approximately matches the brown trout reproductive period in Italy, should likely imply waters cold enough to ensure spawning and embryological stages growth (estimated as 1.9°C–13°C; MacCrimmon and Marshall 1968). Similarly, the maximum temperature of the warmest month (BIO5 contribution = 18.2% and 15.2%) negatively influences trout presence and survival, as water temperatures should exceed the thermal tolerance range, that is 6.5°C–15.8°C for *S. trutta* (according to FishBase; https://www.fishbase.de/summary/SpeciesSummary.php?ID=238&AT=Salmo%20trutta) and approximatively 10°C–17°C for Italian populations (Zanetti 2016).

Also, precipitations of the driest month appear quite critical (BIO14 importance = 11.9% and 20.0%) to express an adequate Salmonid habitat, likely because droughts and dry riverbeds should occur during such a month, hence preventing wild population survival. Annual precipitation, which is known to affect brown trout distribution across European watercourses (Filipe et al. 2013), interestingly showed lower importance compared to the precipitation of the driest month in our modelling framework, suggesting the latter has stronger predictiveness and possibly more biological relevance.

The relevant, yet non‐primary, relative importance of elevation (17.5% and 14.4%, Gini and Permutation, respectively) is quite surprising considering the biased altitudinal distribution of brown trout presence towards medium‐high altitudes (Figures 1 and 2), as well as the crucial role in defining the traditional Salmonid zone. Nevertheless, elevation is a proxy of multiple direct gradients—such as air and water temperatures, precipitation, presence of cold springs, water‐dissolved oxygen and anthropic disturbance (Almodóvar et al. 2012; Carosi et al. 2020; Figure S1)—that may better predict brown trout spatial distribution.

We revealed a negligible role of lithology in influencing brown trout distribution in the study area, despite previous studies providing evidence for an association between permeable lithotypes and the occurrence of native populations (Splendiani et al. 2013, 2016; Lorenzoni et al. 2019). However, the contribution of lithology should not be generalised, being possibly biased by the categorical nature of lithology (as opposed to the five other continuous predictors) and the occurrence in our dataset of both native and stocked populations, which were found to associate with opposite lithotypes (Splendiani et al. 2013, 2016; Lorenzoni et al. 2019).

Lastly, the relative importance of the anthropic disturbance compound index was almost insignificant. Though analogous results emerged from other studies targeting brown trout in Italian and European freshwaters (Filipe et al. 2013; Splendiani et al. 2013, 2016), the explanation remains controversial. Beyond a realistic lack of predictive relevance, we may argue that the native spatial resolution of this layer (1 km) and/or the human pressures from which it relies may not adequately reflect the actual anthropogenic impact on brown trout habitat.

Our study reiterated the importance of modelling approaches as complementary tools for the management of (Mediterranean) brown trout populations and their habitat while providing useful indications to disclose wild populations in infrequent habitat, possibly worthy of protection. Indeed, the Habitat Directive envisages habitat restoration and enhancement also through the identification and monitoring of both native and exotic freshwater fish populations. We contributed to defining the relative importance of environmental and climate predictors to depict brown trout spatial distribution, pointing out the central role of thermal suitability and habitat availability. Besides, our outcomes indirectly suggested that ecologically meaningful bioclimatic constraints (e.g., maximum/minimum values of simple gradients) contributed to the modelling of the spatial distribution of *S. trutta* complex more strongly than multi‐proxy descriptors (e.g., elevation) and more than cumulative values (e.g., annual precipitation). This is likely to be a property of our modelling framework, which relies on a tree‐based method, as the RF is able to effectively deal with predictive variables showing different levels of correlation among them (Figure S1) and with the response variable. Notably, the spatially consistent environmental variables we considered in our model conveniently approximated some crucial (abiotic) features of freshwaters while circumventing their limitations in terms of spatial coverage and/or temporal scale (e.g., punctual and often fragmentary data). These features strengthen the generality of our modelling framework while enhancing its applicability and reproducibility.

Next perspectives would include the on‐field validation of the predicted presences in unexplored but suitable watercourses, the inclusion of population origin information (e.g., native viable, self‐sustainable naturalised and stocked) in the modelling procedure and the implementation of future projections, as global warming was predicted to reduce the suitable available habitat of brown trout in Spain (Almodóvar et al. 2012).

## Author Contributions

**Lorenzo Talarico:** conceptualization (equal), investigation (equal), methodology (equal), visualization (equal), writing – original draft (equal), writing – review and editing (equal). **Elena Catucci:** formal analysis (equal), investigation (equal), methodology (equal), visualization (equal), writing – original draft (equal), writing – review and editing (equal). **Marco Martinoli:** investigation (equal), writing – review and editing (equal). **Michele Scardi:** conceptualization (equal), supervision (equal), writing – review and editing (equal). **Lorenzo Tancioni:** conceptualization (equal), investigation (equal), supervision (equal), writing – review and editing (equal).

## Conflicts of Interest

The authors declare no conflicts of interest.

## Supporting information


**Appendix S1.** Supporting Information.

## Data Availability

The whole dataset used for this study is provided as Table S1.

## References

[ece371658-bib-0001] Almodóvar, A. , G. G. Nicola , D. Ayllón , and B. Elvira . 2012. “Global Warming Threatens the Persistence of Mediterranean Brown Trout.” Global Change Biology 18, no. 5: 1549–1560. 10.1111/j.1365-2486.2011.02608.x.

[ece371658-bib-0002] Aparicio, E. , R. Rocaspana , A. De Sostoa , A. Palau‐Ibars , and C. Alcaraz . 2018. “Movements and Dispersal of Brown Trout ( *Salmo trutta* Linnaeus, 1758) in Mediterranean Streams: Influence of Habitat and Biotic Factors.” PeerJ 6: e5730. 10.7717/peerj.5730. 30345173 PMC6188007

[ece371658-bib-0003] Boulesteix, A.‐L. , S. Janitza , J. Kruppa , and I. R. König . 2012. “Overview of Random Forest Methodology and Practical Guidance With Emphasis on Computational Biology and Bioinformatics.” Wiley Interdisciplinary Reviews: Data Mining and Knowledge Discovery 2, no. 6: 493–507.

[ece371658-bib-0004] Breiman, L. 2001. “Random Forests.” Machine Learning 45: 5–32.

[ece371658-bib-0005] Breiman, L. , J. Friedman , R. Olshen , and C. Stone . 1984. “Classification and Regression Trees.” Group 37, no. 15: 237–251.

[ece371658-bib-0006] Carosi, A. , L. Ghetti , R. Padula , and M. Lorenzoni . 2020. “Population Status and Ecology of the *Salmo trutta* Complex in an Italian River Basin Under Multiple Anthropogenic Pressures.” Ecology and Evolution 10, no. 14: 7320–7333. 10.1002/ece3.6457. 32760531 PMC7391546

[ece371658-bib-0007] Catucci, E. , and M. Scardi . 2022. “Fractal Dimension of *Posidonia oceanica* Meadows for the Assessment of Their Ecological Condition.” Estuarine, Coastal and Shelf Science 274: 107925. 10.1016/j.ecss.2022.107925.

[ece371658-bib-0008] Cianfrani, C. , H. F. Satizábal , and C. Randin . 2015. “A Spatial Modelling Framework for Assessing Climate Change Impacts on Freshwater Ecosystems: Response of Brown Trout ( *Salmo trutta* L.) Biomass to Warming Water Temperature.” Ecological Modelling 313: 1–12. 10.1016/j.ecolmodel.2015.06.023.

[ece371658-bib-0009] Cohen, J. 1960. “Kappa: Coefficient of Concordance.” Educational and Psychological Measurement 20, no. 37: 37–46.

[ece371658-bib-0010] Dawson, T. P. , S. T. Jackson , J. I. House , I. C. Prentice , and G. M. Mace . 2011. “Beyond Predictions: Biodiversity Conservation in a Changing Climate.” Science 332, no. 6025: 53–58. 10.1126/science.1200303. 21454781

[ece371658-bib-0011] Dettinger, M. D. , and H. F. Diaz . 2000. “Global Characteristics of Stream Flow Seasonality and Variability.” Journal of Hydrometeorology 1, no. 4: 289–310. 10.1175/1525-7541(2000)001<0289:GCOSFS>2.0.CO;2.

[ece371658-bib-0012] Fabiani, A. , P. Gratton , I. A. Zappes , et al. 2018. “Investigating the Genetic Structure of Trout From the Garden of Ninfa (Central Italy): Suggestions for Conservation and Management.” Fisheries Management and Ecology 25, no. 1: 1–11. 10.1111/fme.12259.

[ece371658-bib-0013] Filipe, A. F. , D. Markovic , F. Pletterbauer , et al. 2013. “Forecasting Fish Distribution Along Stream Networks: Brown Trout ( *Salmo trutta* ) in Europe.” Diversity and Distributions 19, no. 8: 1059–1071. 10.1111/ddi.12086.

[ece371658-bib-0014] Huet, M. 1959. “Profiles and Biology of Western European Streams as Related to Fish Management.” Transactions of the American Fisheries Society 88, no. 3: 155–163. 10.1577/1548-8659(1959)88[155:PABOWE]2.0.CO;2.

[ece371658-bib-0015] Illies, J. , and L. Botosaneanu . 1963. “Problèmes Et Méthodes de la Classification Et de la Zonation Écologique Des Eaux Courantes, Considerées Surtout du Point de Vue Faunistique: Avec 18 Figures Dans le Texte Et en Supplément.” Internationale Vereinigung für Theoretische Und Angewandte Limnologie: Mitteilungen 12, no. 1: 1–57.

[ece371658-bib-0016] Jonsson, B. , and N. Jonsson . 2018. “Chapter 10 ‐ Habitat as Template for Life‐Histories.” In Brown Trout: Biology, Ecology and Management, edited by J. Lobón‐Cerviá and N. Sanz , 65–102. John Wiley & Sons, Ltd.

[ece371658-bib-0017] Kottelat, M. , and J. Freyhof . 2007. Handbook of European Freshwater Fishes. Publications Kottelat.

[ece371658-bib-0018] Liaw, A. , and M. Wiener . 2002. “Classification and Regression by randomForest.” R News 2, no. 3: 18–22.

[ece371658-bib-0019] Lobón‐Cerviá, J. 2018. “Chapter 1 ‐ Introduction: Princess of the Streams: The Brown Trout *Salmo trutta* L. as Aquatic Royalty.” In Brown Trout: Biology, Ecology and Management, edited by J. Lobón‐Cerviá and N. Sanz , 65–102. John Wiley & Sons, Ltd.

[ece371658-bib-0020] Lorenzoni, M. , A. Carosi , M. Giovannotti , G. La Porta , A. Splendiani , and V. Caputo Barucchi . 2019. “Ecology and Conservation of the Mediterranean Trout in the Central Apennines (Italy).” Journal of Limnology 78, no. 1: 1–13. 10.4081/jlimnol.2018.1806.

[ece371658-bib-0021] Louppe, G. , L. Wehenkel , A. Sutera , and P. Geurts . 2013. “Understanding Variable Importances in Forests of Randomized Trees.” In Advances in Neural Information Processing Systems 26, edited by C. J. C. Burges , L. Bottou , M. Welling , Z. Ghahramani , and K. Q. Weinberger , 431–439. Curran Associates, Inc.

[ece371658-bib-0022] MacCrimmon, H. R. , and T. L. Marshall . 1968. “World Distribution of Brown Trout, *Salmo trutta* .” Journal of the Fisheries Research Board of Canada 25, no. 12: 2527–2548. 10.1139/f68-225.

[ece371658-bib-0023] Mäki‐Petäys, A. , T. Muotka , A. Huusko , P. Tikkanen , and P. Kreivi . 1997. “Seasonal Changes in Habitat Use and Preference by Juvenile Brown Trout, *Salmo trutta* , in a Northern Boreal River.” Canadian Journal of Fisheries and Aquatic Sciences 54: 520–530. 10.1139/f96-311.

[ece371658-bib-0024] Mosher, J. J. , G. C. Klein , A. G. Marshall , and R. H. Findlay . 2010. “Influence of Bedrock Geology on Dissolved Organic Matter Quality in Stream Water.” Organic Geochemistry 41, no. 11: 1177–1188. 10.1016/j.orggeochem.2010.08.004.

[ece371658-bib-0025] Mouton, A. M. , J. D. Alcaraz‐Hernández , B. De Baets , P. L. M. Goethals , and F. Martínez‐Capel . 2011. “Data‐Driven Fuzzy Habitat Suitability Models for Brown Trout in Spanish Mediterranean Rivers.” Environmental Modelling and Software 26, no. 5: 615–622. 10.1016/j.envsoft.2010.12.001.

[ece371658-bib-0026] Muñoz‐Mas, R. , A. Lopez‐Nicolas , F. Martínez‐Capel , and M. Pulido‐Velazquez . 2016. “Shifts in the Suitable Habitat Available for Brown Trout ( *Salmo trutta* L.) Under Short‐Term Climate Change Scenarios.” Science of the Total Environment 544: 686–700. 10.1016/j.scitotenv.2015.11.147. 26674698

[ece371658-bib-0027] Osborne, P. E. , and S. Suárez‐Seoane . 2002. “Should Data Be Partitioned Spatially Before Building Large‐Scale Distribution Models?” Ecological Modelling 157, no. 2: 249–259. 10.1016/S0304-3800(02)00198-9.

[ece371658-bib-0028] Polgar, G. , M. Iaia , T. Righi , and P. Volta . 2022. “The Italian Alpine and Subalpine Trouts: Taxonomy, Evolution, and Conservation.” Biology 11: 576. 10.3390/biology11040576. 35453775 PMC9026872

[ece371658-bib-0029] R Development Core Team . 2022. R: A Language and Environment for Statistical Computing. R Foundation for Statistical Computing. https://www.R‐project.org/.

[ece371658-bib-0030] Rodríguez, J. P. , L. Brotons , J. Bustamante , and J. Seoane . 2007. “The Application of Predictive Modelling of Species Distribution to Biodiversity Conservation.” Diversity and Distributions 13, no. 3: 243–251. 10.1111/j.1472-4642.2007.00356.x.

[ece371658-bib-0031] Rondinini, C. , A. Battistoni , and C. Teofili . 2022. Lista Rossa IUCN Dei Vertebrati Italiani 2022. Comitato Italiano IUCN e Ministero dell'Ambiente e della sicurezza Energetica.

[ece371658-bib-0032] Rossi, A. R. , G. Petrosino , V. Milana , M. Martinoli , A. Rakaj , and L. Tancioni . 2019. “Genetic Identification of Native Populations of Mediterranean Brown Trout *Salmo trutta* L. Complex (Osteichthyes: Salmonidae) in Central Italy.” European Zoological Journal 86, no. 1: 424–431. 10.1080/24750263.2019.1686077.

[ece371658-bib-0033] Rossi, A. R. , L. Talarico , G. Petrosino , S. Crescenzo , and L. Tancioni . 2022. “Conservation Genetics of Mediterranean Brown Trout in Central Italy (Latium): A Multi‐Marker Approach.” Water 14, no. 6: 937. 10.3390/w14060937.

[ece371658-bib-0034] Sarrocco, S. , G. Maio , D. Celauro , and L. Tancioni . 2012. Carta Della Biodiversità Ittica Delle Acque Correnti Del Lazio. ARP.

[ece371658-bib-0035] Splendiani, A. , P. Ruggeri , M. Giovannotti , and V. Caputo Barucchi . 2013. “Role of Environmental Factors in the Spread of Domestic Trout in Mediterranean Streams.” Freshwater Biology 58, no. 10: 2089–2101. 10.1111/fwb.12193.

[ece371658-bib-0036] Splendiani, A. , P. Ruggeri , M. Giovannotti , et al. 2016. “Alien Brown Trout Invasion of the Italian Peninsula: The Role of Geological, Climate and Anthropogenic Factors.” Biological Invasions 18, no. 7: 2029–2044. 10.1007/s10530-016-1149-7.

[ece371658-bib-0037] Tarquini, S. , I. Isola , M. Favalli , A. Battistini , and R. Dotta . 2023. TINITALY, a Digital Elevation Model of Italy With a 10 Meters Cell Size Version 1.1. Istituto Nazionale di Geofisica e Vulcanologia (INGV). 10.13127/tinitaly/1.1.

[ece371658-bib-0038] Thomas, A. L. , E. Dambrine , D. King , J. P. Party , and A. Probst . 1999. “A Spatial Study of the Relationships Between Streamwater Acidity and Geology, Soils and Relief (Vosges, Northeastern France).” Journal of Hydrology 217, no. 1: 35–45. 10.1016/S0022-1694(99)00014-1.

[ece371658-bib-0039] Venter, O. , E. W. Sanderson , A. Magrach , et al. 2016. “Sixteen Years of Change in the Global Terrestrial Human Footprint and Implications for Biodiversity Conservation.” Nature Communications 7, no. 1: 12558. 10.1038/ncomms12558. PMC4996975 27552116

[ece371658-bib-0040] Vinciguerra, D. 1902. “Sulla Presenza di *Salmo macrostigma* Dum. Nelle Paludi Pontine.” Monitore Zoologico Italiano 12: 23–24.

[ece371658-bib-0041] Vismara, R. , A. Azzellino , R. Bosi , G. Crosa , and G. Gentili . 2001. “Habitat Suitability Curves for Brown Trout ( *Salmo trutta* Fario L.) in the River Adda, Northern Italy: Comparing Univariate and Multivariate Approaches.” Regulated Rivers: Research & Management 17, no. 1: 37–50. 10.1002/1099-1646(200101/02)17:1<37::AID-RRR606>3.0.CO;2-Q.

[ece371658-bib-0042] Zaccara, S. , S. Trasforini , C. M. Antognazza , C. Puzzi , J. R. Britton , and G. Crosa . 2015. “Morphological and Genetic Characterization of Sardinian Trout *Salmo cettii* Rafinesque, 1810 and Their Conservation Implications.” Hydrobiologia 760, no. 1: 205–223. 10.1007/s10750-015-2322-1.

[ece371658-bib-0043] Zanetti, M. 2016. “ *S. ghigii*/*S. cettii* .” In Manuali Per il Monitoraggio di Specie e Habitat di Interesse Comunitario (Direttiva 92/43/CEE) in Italia: Specie Animali, edited by F. Stoch and P. Genovesi . ISPRA. Serie Manuali e Linee Guida, 141/2016.

