# Supplementary material for: Modelling Salmo trutta Complex Spatial Distribution in Central Italy: A Random Forest Approach Revealing Underrepresented Lowland Populations Based on Spatially‐Explicit Predictors
Source: Ecol Evol. 2025 Jun 26;15(7):e71658. doi: 10.1002/ece3.71658 (PMC12202777; doi:10.1002/ece3.71658)
Supplement: Supplementary file 1 — Appendix S1. Supporting Information. [file ECE3-15-e71658-s001.docx]

# **Supplementary information for**

“Modelling *Salmo trutta* complex spatial distribution in central Italy: a random forest approach revealing underrepresented lowland populations based on spatially-explicit predictors”

*L. Talarico, E. Catucci, M. Martinoli, M. Scardi, L. Tancioni*

### **Figure S1**

Distribution of observations (N=406) and Pearson’s correlation coefficients (with significance levels: * = p<0.05, ** = p<0.01, *** = p<0.001) for pairs of numeric predictors employed in this study – the graphical output is generated by the *chart.Correlation* function of the *PerformanceAnalytics* R-package.


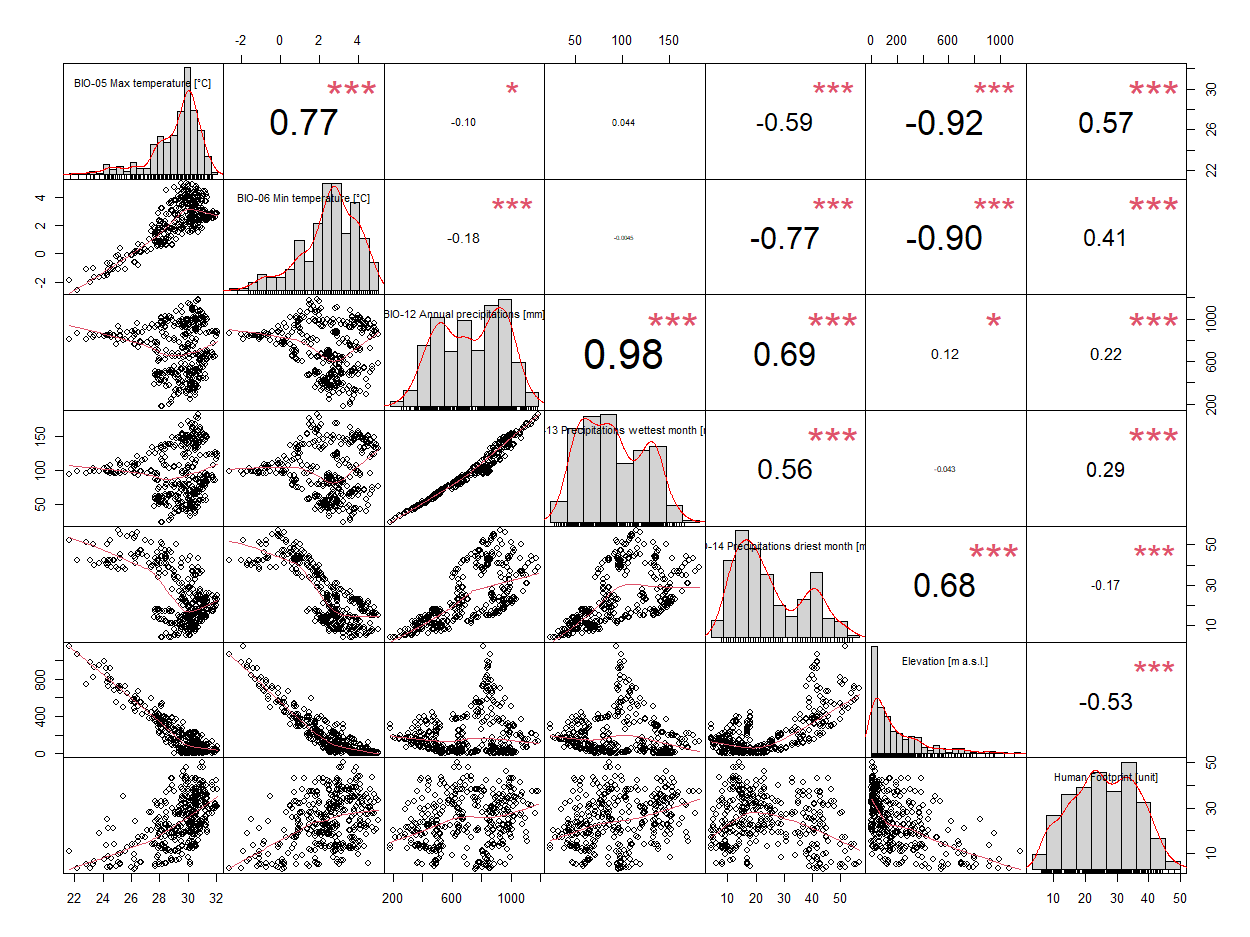


### **Figure S2**

Sixty-three grid cells of size 20x20 km containing 406 brown trout records (red dots = presence, white dots = absence) scattered across four geographic quadrants indicated by different colours.
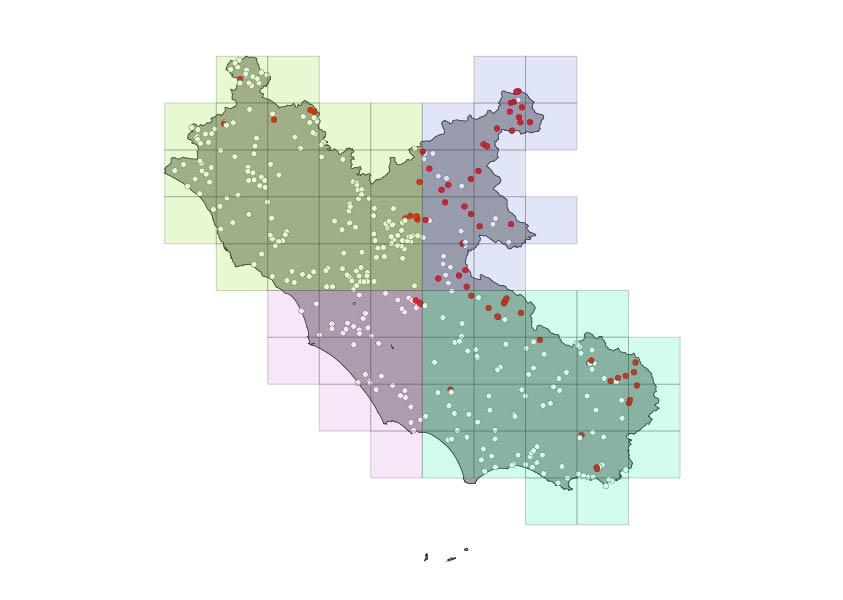


###

### **Figure S3**

The ROC curve analysis for optimal threshold identification, namely the black dot on the red curve with associated sensitivity and specificity values, along with the AUC.


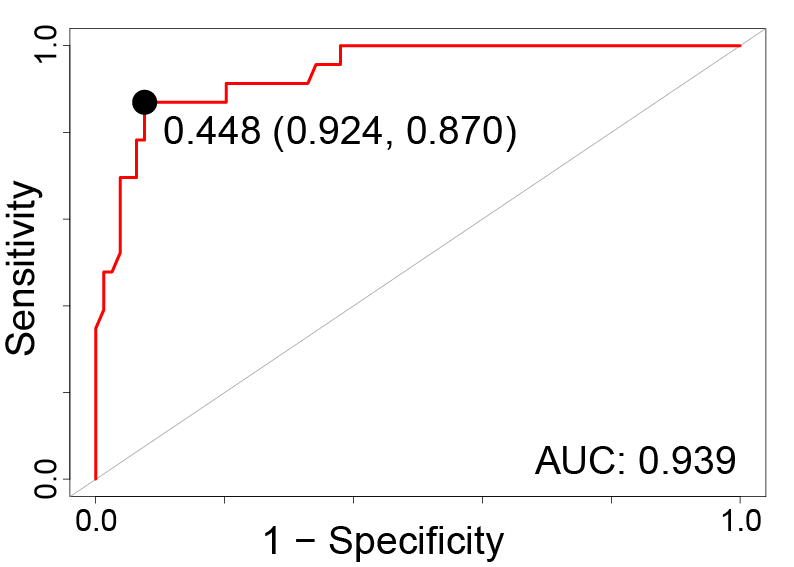


###

### **Table S1**

Complete input dataset: coordinates of 406 *S. trutta* presence/absence data (binary: presence = 1, absence = 0) with corresponding values for environmental/bioclimatic predictors and the anthropogenic disturbance index (HF2009) used in the present study, and the abundance classes (abundance: absence, low-medium density, high density). Site codes in red denote 11 brown trout presences from unpublished data.

| **Site** | **Latitude** | **Longitude** | **Binary** | **Abundance** | **Lithotype** | **BIO_05** | **BIO_06** | **BIO_12** | **BIO_13** | **BIO_14** | **Elevation** | **HF2009** |
| --- | --- | --- | --- | --- | --- | --- | --- | --- | --- | --- | --- | --- |
| AIA1 | 295004.4 | 4687177.4 | 0 | absence | Post-orogenic | 30.9 | 2.5 | 483 | 62 | 16 | 57.87 | 19.12 |
| ALA1 | 350990 | 4614965.3 | 0 | absence | Post-orogenic | 30 | 1.6 | 1148 | 172 | 42 | 212.55 | 37 |
| AMA1 | 357346 | 4594091.5 | 0 | absence | Carbonatic | 30.3 | 2.8 | 1102 | 169 | 31 | 62 | 17.29 |
| ANI1 | 359757.9 | 4638322.2 | 1 | high | Carbonatic | 23.3 | -1 | 830 | 103 | 50 | 814.02 | 5.25 |
| ANI2 | 350164 | 4636396.7 | 1 | high | Carbonatic | 26.1 | -0.1 | 881 | 110 | 55 | 573.12 | 14 |
| Anie1 | 349904.8 | 4636738.7 | 1 | high | Carbonatic | 26.3 | 0 | 889 | 111 | 54 | 545.6 | 14 |
| Anie2 | 346111.3 | 4640325.1 | 1 | high | Carbonatic | 26.2 | 0.1 | 868 | 109 | 53 | 483.1 | 7.53 |
| Anie3 | 336879.7 | 4649380.6 | 1 | high | Arenaceous flysch | 28.2 | 1 | 879 | 113 | 44 | 331.86 | 20 |
| Anie4 | 333567 | 4654191.5 | 1 | high | Carbonatic | 28.5 | 1.1 | 858 | 108 | 43 | 315.45 | 28 |
| Anie5 | 324649.8 | 4652817.1 | 1 | low-medium | Carbonatic | 29 | 1.9 | 798 | 104 | 35 | 254.94 | 37 |
| APA1 | 355835 | 4676036.9 | 1 | high | Clay flysch | 24.1 | -1.6 | 840 | 103 | 42 | 990.79 | 7.68 |
| Arro1 | 277454.4 | 4657251.7 | 0 | absence | Volcanic | 30.5 | 3.4 | 343 | 45 | 7 | 130.9 | 33 |
| Arro2 | 276595.5 | 4656216.7 | 0 | absence | Volcanic | 30.6 | 3.5 | 366 | 48 | 8 | 99.2 | 37 |
| Arro3 | 276920.7 | 4649290.4 | 0 | absence | Post-orogenic | 30.2 | 3.5 | 470 | 62 | 8 | 40.14 | 24.3 |
| Arro4 | 272426.7 | 4642012.2 | 0 | absence | Post-orogenic | 29.8 | 4 | 574 | 76 | 9 | 17.57 | 32.01 |
| C.I. | 390353 | 4617268.5 | 0 | absence | Clay flysch | 28.4 | 0.9 | 985 | 135 | 44 | 289.06 | 25 |
| CadPe | 269483.7 | 4653726.1 | 0 | absence | Volcanic | 30.1 | 3.9 | 332 | 44 | 5 | 113.61 | 22.37 |
| CadTr | 271377.6 | 4655531.8 | 0 | absence | Volcanic | 30.2 | 3.8 | 282 | 36 | 5 | 152.5 | 29.34 |
| CAM | 352944 | 4635229.2 | 0 | absence | Carbonatic | 25.7 | -0.1 | 875 | 109 | 54 | 643.16 | 12.73 |
| CAM1 | 291835 | 4690761.1 | 0 | absence | Post-orogenic | 30.8 | 2.4 | 442 | 56 | 15 | 45.58 | 39.73 |
| CAN1 | 316539.2 | 4693972.7 | 1 | high | Carbonatic | 28 | 0.9 | 607 | 75 | 36 | 416.02 | 18.85 |
| CAR1 | 390043.6 | 4617726.3 | 0 | absence | Clay flysch | 28.5 | 1 | 989 | 137 | 43 | 291.29 | 22.73 |
| CARP | 390008 | 4617756.8 | 1 | high | Clay flysch | 28.5 | 1 | 989 | 137 | 43 | 292.2 | 22.73 |
| CAS1 | 340468.7 | 4622347 | 0 | absence | Post-orogenic | 31 | 1.8 | 1064 | 156 | 37 | 190.77 | 41 |
| CON1 | 383997.8 | 4625230.3 | 0 | absence | Clay flysch | 28.1 | 0.5 | 1016 | 137 | 48 | 312.92 | 30.73 |
| COR1 | 318130.2 | 4670384.9 | 1 | low-medium | Post-orogenic | 29.4 | 1.9 | 647 | 80 | 31 | 246.09 | 27 |
| COR2 | 308834.3 | 4673378.9 | 0 | absence | Post-orogenic | 31.3 | 2.5 | 651 | 86 | 21 | 74.02 | 22.45 |
| COR3 | 307354.3 | 4671287.9 | 0 | absence | Post-orogenic | 31.5 | 2.5 | 650 | 87 | 20 | 46.41 | 29.36 |
| COR4 | 305601.3 | 4669390.9 | 0 | absence | Post-orogenic | 31.8 | 2.5 | 668 | 89 | 19 | 26.24 | 38 |
| COS1 | 361995.3 | 4623091.6 | 0 | absence | Carbonatic | 29.2 | 1 | 1068 | 147 | 50 | 370.55 | 27.63 |
| COS2 | 365250.9 | 4625976.3 | 0 | absence | Carbonatic | 26.4 | 0.2 | 961 | 120 | 56 | 598.67 | 5.3 |
| COS3 | 365311.9 | 4614640.3 | 0 | absence | Post-orogenic | 30.2 | 1.5 | 1138 | 168 | 44 | 220.56 | 31 |
| COS4 | 363199.9 | 4611953.4 | 0 | absence | Post-orogenic | 30.7 | 1.5 | 1172 | 176 | 43 | 197.42 | 37 |
| COS5 | 360274.9 | 4605441.4 | 0 | absence | Post-orogenic | 30.7 | 1.9 | 1188 | 182 | 39 | 125.46 | 37.34 |
| COSA | 367867.5 | 4626683.1 | 1 | high | Carbonatic | 25.1 | -0.1 | 930 | 115 | 57 | 695.79 | 6.26 |
| Cpap1 | 313479.4 | 4643419.7 | 0 | absence | Volcanic | 31.9 | 2.8 | 874 | 123 | 24 | 56.8 | 30.73 |
| Cpap2 | 311908.3 | 4644531.6 | 0 | absence | Volcanic | 32.1 | 2.9 | 883 | 125 | 23 | 38.19 | 38 |
| Cvca1 | 288360.3 | 4632487.7 | 0 | absence | Post-orogenic | 30 | 3.8 | 745 | 103 | 14 | 13.22 | 34.66 |
| DIL1 | 353426 | 4624874.3 | 0 | absence | Carbonatic | 28.2 | 0.7 | 943 | 124 | 50 | 543.79 | 14.3 |
| ELI1 | 379251.8 | 4612829.4 | 0 | absence | Carbonatic | 29.6 | 1.4 | 1066 | 153 | 43 | 222.16 | 35 |
| FAR1 | 321005.2 | 4677718.9 | 0 | absence | Post-orogenic | 29.3 | 1.5 | 678 | 84 | 34 | 278.54 | 28 |
| FAR2 | 319285.2 | 4678004.9 | 1 | high | Post-orogenic | 29.1 | 1.6 | 645 | 80 | 33 | 245.12 | 28 |
| FAR3 | 315800.2 | 4677903.9 | 1 | low-medium | Post-orogenic | 29.7 | 1.9 | 640 | 80 | 29 | 200.27 | 19.32 |
| FAR5 | 309569.3 | 4676707.9 | 0 | absence | Post-orogenic | 30.9 | 2.4 | 634 | 81 | 23 | 79.8 | 22.8 |
| FAR6 | 305738.3 | 4675970.9 | 0 | absence | Post-orogenic | 31.3 | 2.5 | 616 | 81 | 20 | 48.99 | 31.42 |
| FAR7 | 305025.1 | 4675752.9 | 0 | absence | Post-orogenic | 31.2 | 2.5 | 604 | 79 | 20 | 66.96 | 31.42 |
| FAR8 | 312659.9 | 4679656.3 | 1 | low-medium | Carbonatic | 30.1 | 2.2 | 633 | 80 | 26 | 122.85 | 19.32 |
| FAR9 | 315180.3 | 4679241.7 | 1 | low-medium | Post-orogenic | 29.8 | 2 | 644 | 80 | 28 | 189.88 | 19.32 |
| FDA1 | 392098.7 | 4596644.5 | 0 | absence | Post-orogenic | 30 | 2.2 | 988 | 146 | 33 | 113.84 | 41 |
| FDA2 | 395186.7 | 4585244.6 | 0 | absence | Post-orogenic | 30 | 2.7 | 956 | 146 | 28 | 38.56 | 33 |
| FDM1 | 342264.1 | 4675213.9 | 1 | low-medium | Arenaceous flysch | 26 | -0.1 | 809 | 101 | 45 | 561.63 | 4.28 |
| FIB1 | 391342.5 | 4616576.9 | 0 | absence | Carbonatic | 28.4 | 0.9 | 982 | 135 | 43 | 294 | 22.73 |
| FIB2 | 389037.7 | 4616826.3 | 1 | high | Clay flysch | 28.5 | 1 | 993 | 137 | 44 | 292.74 | 32 |
| FON1 | 390198.7 | 4613976.4 | 0 | absence | Carbonatic | 28.1 | 0.9 | 979 | 134 | 44 | 324.87 | 22.73 |
| FON2 | 389277.7 | 4616701.3 | 1 | low-medium | Clay flysch | 28.5 | 1 | 993 | 137 | 44 | 292.61 | 32 |
| FQA1 | 385705.8 | 4585878.6 | 1 | high | Post-orogenic | 29.6 | 2.4 | 995 | 151 | 31 | 58.9 | 18.4 |
| GAL1 | 305440.3 | 4682786.8 | 0 | absence | Post-orogenic | 30.8 | 2.2 | 587 | 76 | 21 | 57.13 | 22.42 |
| GOL1 | 384856.8 | 4623487.3 | 0 | absence | Clay flysch | 28.7 | 1.1 | 1024 | 139 | 47 | 301.57 | 38 |
| LAC1 | 390347.7 | 4622132.3 | 0 | absence | Carbonatic | 27 | 0.3 | 926 | 117 | 52 | 505.73 | 17.37 |
| LAC2 | 385330.8 | 4620816.3 | 0 | absence | Clay flysch | 28.8 | 1.2 | 1013 | 138 | 46 | 290.08 | 43 |
| LIR1 | 384216.8 | 4624346.3 | 1 | low-medium | Clay flysch | 28.4 | 0.8 | 1023 | 139 | 47 | 294.22 | 33 |
| LIR4 | 376418.8 | 4605003.4 | 0 | absence | Post-orogenic | 30.3 | 1.8 | 1079 | 161 | 37 | 109.1 | 22.63 |
| LIR5 | 390382.5 | 4587179.1 | 0 | absence | Post-orogenic | 30.2 | 2.6 | 973 | 148 | 29 | 49.35 | 23 |
| LT001 | 398602.2 | 4566541.5 | 0 | absence | Post-orogenic | 29.9 | 3.6 | 921 | 142 | 22 | 6.31 | 41.41 |
| LT002 | 397408 | 4566952 | 0 | absence | Post-orogenic | 29.8 | 3.7 | 922 | 141 | 22 | 6.37 | 35.8 |
| LT003 | 394653.4 | 4573071.6 | 0 | absence | Clay flysch | 29.7 | 3.3 | 954 | 144 | 25 | 46.74 | 29.01 |
| LT004 | 404648 | 4571220.3 | 0 | absence | Post-orogenic | 29.6 | 3.5 | 922 | 139 | 24 | 12.27 | 13.27 |
| LT006 | 392945.6 | 4572780 | 0 | absence | Clay flysch | 29.4 | 3.2 | 982 | 147 | 27 | 55.51 | 29.02 |
| LT007 | 390765.3 | 4568128 | 0 | absence | Post-orogenic | 29.8 | 3.8 | 941 | 145 | 23 | 13.82 | 35 |
| LT008 | 379796.2 | 4567928.7 | 0 | absence | Carbonatic | 29.5 | 3.8 | 953 | 146 | 23 | 21.31 | 38.79 |
| LT009 | 363764.1 | 4576758.8 | 0 | absence | Post-orogenic | 30.3 | 4.1 | 985 | 152 | 21 | 2.85 | 20.83 |
| LT010 | 361826.7 | 4573363.1 | 0 | absence | Post-orogenic | 30.1 | 4.4 | 950 | 148 | 19 | 1.5 | 22.21 |
| LT011 | 364707.7 | 4578705.3 | 0 | absence | Post-orogenic | 30.5 | 4 | 1006 | 154 | 22 | 5.05 | 37.01 |
| LT012 | 364393.3 | 4578323.8 | 0 | absence | Post-orogenic | 30.5 | 4 | 1006 | 154 | 22 | 4.1 | 37.01 |
| LT013 | 358698.1 | 4577460.9 | 0 | absence | Carbonatic | 29.9 | 4.5 | 1001 | 152 | 22 | 12.22 | 27.16 |
| LT014 | 356890.1 | 4573769 | 0 | absence | Post-orogenic | 30 | 4.8 | 958 | 148 | 19 | 8.03 | 19.79 |
| LT015 | 357076.7 | 4594740.1 | 0 | absence | Carbonatic | 30.3 | 2.9 | 1109 | 170 | 31 | 63.9 | 15.53 |
| LT016 | 349030.6 | 4588071.8 | 0 | absence | Post-orogenic | 30.7 | 4 | 1035 | 157 | 23 | 17.49 | 34.44 |
| LT017 | 348636 | 4572989.9 | 0 | absence | Post-orogenic | 30.6 | 4.8 | 920 | 140 | 16 | 2.33 | 32.28 |
| LT018 | 348948.1 | 4584157.5 | 0 | absence | Post-orogenic | 30.9 | 4.1 | 1007 | 152 | 21 | 9.44 | 20.76 |
| LT019 | 337624.1 | 4594810.4 | 0 | absence | Post-orogenic | 31.1 | 4.1 | 1020 | 153 | 23 | 15.43 | 34.53 |
| LT020 | 341397.5 | 4591749.9 | 0 | absence | Post-orogenic | 31 | 4.1 | 1014 | 152 | 22 | 5.72 | 24 |
| LT021 | 343886.8 | 4580176.9 | 0 | absence | Post-orogenic | 30.8 | 4.3 | 946 | 144 | 18 | 2.52 | 23 |
| LT022 | 337878.6 | 4586674.6 | 0 | absence | Post-orogenic | 31.1 | 4.2 | 969 | 146 | 20 | 2.13 | 33 |
| LT023 | 329331.3 | 4604916.4 | 1 | high | Post-orogenic | 31.2 | 3.7 | 1025 | 152 | 24 | 24.38 | 26.4 |
| LT024 | 329098.7 | 4598624.2 | 0 | absence | Post-orogenic | 31.4 | 4 | 995 | 148 | 22 | 11.57 | 32 |
| LT025 | 332771.7 | 4587848.7 | 0 | absence | Post-orogenic | 31.2 | 4.4 | 954 | 144 | 19 | 10.93 | 28.73 |
| LT026 | 343052.2 | 4575598.6 | 0 | absence | Post-orogenic | 30.8 | 4.5 | 918 | 140 | 16 | 4.72 | 24.07 |
| LT027 | 328770.4 | 4583693.9 | 0 | absence | Post-orogenic | 30.3 | 4.9 | 893 | 138 | 16 | 6.4 | 34.22 |
| LT028 | 366411 | 4571122 | 0 | absence | Post-orogenic | 30.1 | 4.1 | 948 | 148 | 20 | 11.48 | 15.54 |
| LT029 | 330835.8 | 4583903.1 | 0 | absence | Post-orogenic | 30.8 | 4.5 | 926 | 142 | 17 | 18.35 | 30.14 |
| LT030 | 317228.9 | 4592374.2 | 0 | absence | Post-orogenic | 30.5 | 4.7 | 901 | 137 | 16 | 14.36 | 27.7 |
| LT031 | 323826.1 | 4594055.3 | 0 | absence | Post-orogenic | 31.1 | 4.4 | 945 | 142 | 19 | 16.02 | 36.01 |
| LT032 | 312862.3 | 4598041.3 | 0 | absence | Post-orogenic | 30.5 | 4.5 | 914 | 136 | 17 | 20.72 | 34 |
| LT033 | 329320.5 | 4603792.5 | 0 | absence | Post-orogenic | 31.3 | 3.7 | 1018 | 151 | 24 | 23.8 | 26.4 |
| LT034 | 310563.4 | 4602004 | 0 | absence | Post-orogenic | 30.6 | 4.3 | 912 | 134 | 17 | 38.11 | 31 |
| LT035 | 312999.2 | 4591312 | 0 | absence | Post-orogenic | 30 | 4.9 | 878 | 133 | 16 | 8.01 | 20.73 |
| LT036 | 295292.7 | 4608799.4 | 0 | absence | Post-orogenic | 29.8 | 4.7 | 855 | 124 | 15 | 15.88 | 28.62 |
| LT037 | 295387.2 | 4608284.5 | 0 | absence | Post-orogenic | 29.8 | 4.7 | 855 | 124 | 15 | 22.08 | 28.62 |
| LT038 | 300226.5 | 4611177 | 0 | absence | Volcanic | 30.1 | 4.2 | 868 | 125 | 17 | 47.17 | 30 |
| LT040 | 384554.3 | 4569232 | 1 | low-medium | Post-orogenic | 29.7 | 3.8 | 966 | 147 | 23 | 32.9 | 45.79 |
| LT041 | 377263.7 | 4572614.7 | 0 | absence | Carbonatic | 28.9 | 3 | 988 | 149 | 28 | 192.16 | 40.02 |
| LT042 | 375750.5 | 4565553.8 | 0 | absence | Carbonatic | 29.7 | 4.4 | 929 | 146 | 20 | 41.65 | 18.44 |
| LT043 | 369801.1 | 4569584.9 | 0 | absence | Carbonatic | 29.3 | 4.1 | 948 | 148 | 22 | 90.67 | 27.07 |
| LT044 | 369346 | 4577895.7 | 0 | absence | Post-orogenic | 30.6 | 3.9 | 1001 | 153 | 23 | 8.07 | 43 |
| LT045 | 367018.1 | 4574331.2 | 0 | absence | Post-orogenic | 30.4 | 3.8 | 973 | 150 | 21 | 6.39 | 21.46 |
| LT046 | 348893.7 | 4577210.6 | 0 | absence | Post-orogenic | 30.6 | 4.6 | 952 | 144 | 18 | 7.39 | 33.07 |
| LT047 | 353419.4 | 4575588.6 | 0 | absence | Post-orogenic | 29.9 | 4.5 | 984 | 150 | 21 | 52.5 | 30.14 |
| LT048 | 324568.8 | 4604286.6 | 0 | absence | Post-orogenic | 31 | 3.7 | 988 | 147 | 23 | 43.56 | 39 |
| LT049 | 310363 | 4605020.2 | 0 | absence | Volcanic | 30.7 | 4.1 | 914 | 134 | 18 | 51.43 | 22.53 |
| LT050 | 322945.4 | 4617072.6 | 0 | absence | Volcanic | 30.4 | 3.1 | 949 | 138 | 28 | 220.43 | 14.29 |
| LT051 | 346095.7 | 4602719.4 | 0 | absence | Carbonatic | 27.5 | 2 | 947 | 134 | 40 | 377.33 | 14 |
| LT052 | 346646.3 | 4596363.7 | 0 | absence | Carbonatic | 30.9 | 3.6 | 1084 | 164 | 27 | 70.77 | 33 |
| LT053 | 305126.1 | 4607371.1 | 0 | absence | Volcanic | 30.5 | 4.2 | 897 | 130 | 18 | 62.75 | 32 |
| LT054 | 331517.4 | 4595156.7 | 0 | absence | Post-orogenic | 31.3 | 4.2 | 985 | 148 | 21 | 8.29 | 34 |
| LT055 | 392158.6 | 4572084.1 | 0 | absence | Clay flysch | 29.4 | 3.2 | 982 | 147 | 27 | 48.71 | 29.02 |
| LT056 | 368203.8 | 4580263.3 | 0 | absence | Post-orogenic | 30.4 | 3.7 | 1020 | 155 | 24 | 7.66 | 35 |
| LT057 | 366384.5 | 4578507.9 | 0 | absence | Post-orogenic | 30.6 | 3.9 | 1005 | 153 | 23 | 1.22 | 39.01 |
| LT058 | 298067.3 | 4605410.1 | 0 | absence | Post-orogenic | 29.7 | 4.8 | 843 | 123 | 15 | 20.85 | 27.52 |
| LT059 | 308515.7 | 4602916.1 | 0 | absence | Post-orogenic | 30.5 | 4.3 | 901 | 132 | 17 | 48.31 | 35 |
| LT060 | 344505.1 | 4570194.3 | 0 | absence | Post-orogenic | 30.6 | 4.8 | 882 | 134 | 15 | 3.77 | 35.79 |
| LT062 | 313917.7 | 4588007.5 | 0 | absence | Post-orogenic | 29.9 | 5 | 858 | 133 | 15 | 2.2 | 24.42 |
| Luc1 | 373768.8 | 4606779.4 | 0 | absence | Post-orogenic | 30.3 | 1.6 | 1095 | 164 | 39 | 147.37 | 25.53 |
| MEL1 | 408186.5 | 4612859.9 | 1 | high | Carbonatic | 22.8 | -1.1 | 810 | 95 | 51 | 745.31 | 5.42 |
| MEL2 | 404483.6 | 4611400.4 | 1 | high | Clay flysch | 26.8 | 0.6 | 876 | 115 | 45 | 422.85 | 17.63 |
| MEL3 | 398093.7 | 4609206.4 | 1 | high | Clay flysch | 27.8 | 0.9 | 944 | 131 | 41 | 321.59 | 32 |
| MEL4 | 388138.7 | 4602579.4 | 0 | absence | Post-orogenic | 28.6 | 1.5 | 977 | 139 | 39 | 166.61 | 11.85 |
| MELF | 408971.2 | 4616935.6 | 1 | high | Carbonatic | 21.6 | -1.9 | 811 | 94 | 52 | 1153.81 | 11.25 |
| MER1 | 372329.9 | 4599614.5 | 0 | absence | Post-orogenic | 30.4 | 2 | 1084 | 164 | 35 | 115.41 | 33 |
| Mign1 | 259400.3 | 4671541.3 | 0 | absence | Volcanic | 28.1 | 3.2 | 180 | 24 | 4 | 266.67 | 12.34 |
| Mign2 | 257209.8 | 4668474.9 | 0 | absence | Volcanic | 28.8 | 3.6 | 217 | 29 | 4 | 161.21 | 17 |
| Mign3 | 258044.5 | 4668883.9 | 0 | absence | Volcanic | 28.7 | 3.6 | 200 | 27 | 4 | 172.79 | 20 |
| Mign4 | 256691.4 | 4668618.9 | 0 | absence | Volcanic | 28.8 | 3.7 | 244 | 33 | 4 | 157.98 | 17 |
| Mign5 | 256395 | 4668765 | 0 | absence | Volcanic | 28.8 | 3.7 | 244 | 33 | 4 | 151.13 | 17 |
| Mign6 | 254078.2 | 4669546.9 | 0 | absence | Allochthonous flysch | 29.1 | 3.8 | 327 | 43 | 5 | 121.85 | 20 |
| Mign7 | 243766.1 | 4680085.3 | 0 | absence | Allochthonous flysch | 29.9 | 4 | 481 | 63 | 8 | 44.9 | 12.76 |
| Mlen1 | 255126.7 | 4666912.6 | 0 | absence | Volcanic | 29 | 3.8 | 280 | 37 | 5 | 149.24 | 17 |
| Mlen2 | 253623.9 | 4669847.5 | 0 | absence | Allochthonous flysch | 29.1 | 3.8 | 327 | 43 | 5 | 117.86 | 16 |
| MOL1 | 409303.6 | 4607269.7 | 1 | low-medium | Clay flysch | 25.2 | 0.4 | 836 | 108 | 45 | 672.24 | 10 |
| MOL2 | 400574.6 | 4608736.4 | 0 | absence | Clay flysch | 27.3 | 1 | 909 | 125 | 41 | 372.94 | 28 |
| MON1 | 315392.3 | 4679364.8 | 1 | low-medium | Post-orogenic | 29.8 | 2 | 644 | 80 | 28 | 189.04 | 19.32 |
| NER1 | 395309.7 | 4610101.4 | 1 | low-medium | Clay flysch | 28.1 | 0.9 | 966 | 134 | 41 | 313.08 | 20.34 |
| PAG1 | 334356.1 | 4673287.9 | 1 | low-medium | Carbonatic | 26.2 | 0.1 | 760 | 96 | 44 | 545.22 | 3.28 |
| Paliano | 333634.7 | 4626049.4 | 0 | absence | Post-orogenic | 30.6 | 1.8 | 989 | 143 | 35 | 222.98 | 35 |
| PESC | 355578.6 | 4727897.5 | 1 | high | Arenaceous flysch | 24.4 | -1.3 | 846 | 98 | 41 | 797.78 | 9 |
| PET1 | 335074.1 | 4667592.9 | 1 | low-medium | Carbonatic | 26.6 | 0.1 | 781 | 98 | 47 | 551.84 | 6.3 |
| RAP1 | 405866.6 | 4599784.5 | 1 | high | Post-orogenic | 29 | 2.2 | 935 | 134 | 34 | 107.23 | 19 |
| RAP2 | 403083.6 | 4593029.5 | 0 | absence | Post-orogenic | 30.1 | 2.6 | 944 | 141 | 29 | 35.07 | 32 |
| RAPI | 406434.8 | 4601089.1 | 1 | high | Post-orogenic | 28.4 | 2.1 | 929 | 131 | 36 | 147.61 | 14.34 |
| RFiu1 | 249096.3 | 4663171.4 | 0 | absence | Allochthonous flysch | 28.5 | 4.5 | 363 | 48 | 6 | 118.67 | 5.78 |
| Rfiu2 | 245909.3 | 4658693.4 | 0 | absence | Post-orogenic | 29.5 | 5 | 579 | 77 | 8 | 10.06 | 33.28 |
| RI001 | 358441.4 | 4732877.1 | 1 | low-medium | Arenaceous flysch | 25.2 | -0.7 | 843 | 95 | 43 | 695.87 | 5.63 |
| RI002 | 358527 | 4729043.8 | 1 | low-medium | Arenaceous flysch | 24.5 | -1.2 | 843 | 96 | 42 | 918.6 | 4.34 |
| RI003 | 356914.4 | 4728248.9 | 1 | high | Arenaceous flysch | 24.7 | -1.1 | 848 | 97 | 42 | 724.5 | 21 |
| RI004 | 363659.8 | 4719737.4 | 1 | low-medium | Arenaceous flysch | 22.2 | -2.6 | 861 | 99 | 42 | 1069.27 | 3.68 |
| RI005 | 359795.5 | 4719473.9 | 1 | low-medium | Arenaceous flysch | 23.7 | -1.7 | 844 | 98 | 41 | 950.61 | 19 |
| RI006 | 356111.2 | 4716006.2 | 1 | high | Arenaceous flysch | 23.4 | -1.7 | 844 | 99 | 40 | 921.9 | 6.63 |
| RI007 | 360313.9 | 4725946.5 | 1 | low-medium | Arenaceous flysch | 23.1 | -2 | 856 | 98 | 42 | 983.24 | 3.85 |
| RI008 | 355041.2 | 4724093.8 | 1 | high | Arenaceous flysch | 24.2 | -1.4 | 842 | 98 | 41 | 916.04 | 13.73 |
| RI009 | 359161 | 4721801.7 | 1 | high | Arenaceous flysch | 24.3 | -1.5 | 843 | 98 | 40 | 843.29 | 25 |
| RI010 | 358242.7 | 4732526.5 | 1 | high | Arenaceous flysch | 24.8 | -0.9 | 845 | 96 | 42 | 680.46 | 14 |
| RIA1 | 310540.3 | 4677583.9 | 0 | absence | Post-orogenic | 30.9 | 2.4 | 649 | 83 | 23 | 99.39 | 21.66 |
| RMF1 | 384947 | 4584784.7 | 0 | absence | Post-orogenic | 30 | 2.7 | 994 | 151 | 29 | 71.24 | 8.28 |
| RNC1 | 336267.1 | 4668316.9 | 1 | low-medium | Carbonatic | 26.6 | 0.2 | 789 | 98 | 47 | 548.61 | 6.28 |
| RTor1 | 293452.9 | 4611866.3 | 0 | absence | Volcanic | 29.8 | 4.6 | 831 | 119 | 15 | 24.49 | 30.41 |
| SA1 | 312832.3 | 4679729.8 | 1 | low-medium | Carbonatic | 30.1 | 2.2 | 633 | 80 | 26 | 124.39 | 19.32 |
| SAC1 | 334631.1 | 4628234.2 | 0 | absence | Post-orogenic | 30.4 | 1.7 | 995 | 142 | 37 | 228.11 | 21.68 |
| SAC2 | 349914 | 4613990.3 | 0 | absence | Post-orogenic | 29.5 | 1.7 | 1120 | 167 | 41 | 148.77 | 31.63 |
| SAC3 | 361804.9 | 4602779.4 | 0 | absence | Post-orogenic | 30.6 | 2 | 1163 | 178 | 37 | 123.24 | 27.53 |
| SAC4 | 369386.7 | 4598772.8 | 0 | absence | Post-orogenic | 30.5 | 2.1 | 1104 | 168 | 35 | 99.52 | 19.34 |
| Sacc1 | 334960.7 | 4633598.1 | 0 | absence | Post-orogenic | 29.5 | 1.8 | 976 | 135 | 39 | 249.06 | 29.42 |
| Sacc2 | 337111.4 | 4621907.4 | 0 | absence | Post-orogenic | 30.6 | 2 | 1035 | 152 | 35 | 196.15 | 32 |
| Sacc3 | 341202.2 | 4619650.1 | 0 | absence | Post-orogenic | 30.9 | 1.9 | 1076 | 160 | 36 | 175.52 | 36.73 |
| SAL1 | 354865 | 4668374.9 | 1 | low-medium | Carbonatic | 26.5 | -0.8 | 872 | 109 | 46 | 671.36 | 15.63 |
| SAL2 | 347958 | 4676108.9 | 0 | absence | Arenaceous flysch | 26.2 | -0.6 | 841 | 105 | 44 | 578.86 | 9.73 |
| SAL3 | 335785.1 | 4683758.8 | 1 | high | Carbonatic | 26.3 | 0.2 | 763 | 96 | 41 | 450.31 | 3.53 |
| SAL4 | 328927.1 | 4692886.7 | 1 | high | Post-orogenic | 27.9 | 0.6 | 705 | 86 | 42 | 418.54 | 12.42 |
| Sang1 | 263078.1 | 4651110.3 | 0 | absence | Post-orogenic | 30 | 4.4 | 437 | 58 | 6 | 59.79 | 27.02 |
| Scann | 326585.7 | 4636155.2 | 0 | absence | Carbonatic | 27.4 | 1.6 | 766 | 99 | 39 | 596.52 | 20.37 |
| SCR1 | 392157.2 | 4572083.6 | 1 | high | Clay flysch | 29.4 | 3.2 | 982 | 147 | 27 | 48.71 | 29.02 |
| SCR2 | 392482.2 | 4571412.6 | 1 | high | Post-orogenic | 29.6 | 3.4 | 973 | 146 | 26 | 43.23 | 34.11 |
| SEC1 | 403017.6 | 4600872.5 | 0 | absence | Post-orogenic | 29.1 | 2.3 | 959 | 137 | 33 | 88.88 | 27 |
| SET1 | 401340.6 | 4610525.4 | 1 | low-medium | Clay flysch | 27.7 | 0.9 | 920 | 125 | 41 | 368.35 | 24 |
| SIM1 | 353680.3 | 4644287.9 | 1 | high | Carbonatic | 24.5 | -0.7 | 865 | 108 | 52 | 787.73 | 4.25 |
| SIM2 | 352644 | 4642309.8 | 1 | high | Carbonatic | 25.2 | -0.4 | 886 | 111 | 55 | 696.45 | 5.26 |
| Smeil | 340920.4 | 4618485.3 | 0 | absence | Post-orogenic | 30.7 | 1.9 | 1069 | 159 | 37 | 185.28 | 35.34 |
| Ssavo | 331683.6 | 4625454.3 | 0 | absence | Post-orogenic | 30.5 | 2 | 991 | 144 | 33 | 230.74 | 43 |
| SVIT | 316547.4 | 4642260.8 | 1 | high | Volcanic | 31.3 | 2.8 | 865 | 122 | 26 | 134.48 | 23.68 |
| T.AMA1 | 369898.9 | 4620768.3 | 0 | absence | Carbonatic | 28.1 | 0.8 | 1004 | 133 | 51 | 508.94 | 17.31 |
| T.AMA2 | 376263.8 | 4610246.4 | 0 | absence | Carbonatic | 29.6 | 1.4 | 1061 | 154 | 43 | 196.8 | 26.76 |
| T4co1 | 296881.5 | 4679618.4 | 0 | absence | Post-orogenic | 30.4 | 2.6 | 489 | 64 | 16 | 95.14 | 28.13 |
| T4co2 | 297333.4 | 4682678.3 | 0 | absence | Post-orogenic | 30.5 | 2.6 | 498 | 65 | 16 | 64.71 | 26.47 |
| T7ba1 | 297286.7 | 4651600.6 | 0 | absence | Post-orogenic | 31.4 | 2.8 | 669 | 92 | 16 | 34.76 | 34.31 |
| T7ba2 | 294338.7 | 4651599.3 | 0 | absence | Post-orogenic | 31.1 | 3 | 650 | 88 | 15 | 19.36 | 47.79 |
| Taac1 | 296096 | 4628229.2 | 0 | absence | Volcanic | 30.6 | 3.2 | 807 | 115 | 17 | 84.41 | 38.85 |
| Taac2 | 290919.5 | 4630961.9 | 0 | absence | Volcanic | 30 | 3.6 | 759 | 105 | 15 | 36.94 | 38.22 |
| Tabag | 336202.4 | 4656374.1 | 1 | low-medium | Carbonatic | 27.4 | 0.3 | 805 | 100 | 51 | 447.07 | 28 |
| Tacan | 341646.9 | 4644509.1 | 0 | absence | Arenaceous flysch | 28.1 | 0.7 | 909 | 116 | 48 | 532.76 | 33 |
| Tacon | 338581.1 | 4645546.9 | 1 | low-medium | Arenaceous flysch | 28.4 | 0.9 | 919 | 119 | 45 | 344.66 | 21 |
| TAfi1 | 330500 | 4647440.6 | 0 | absence | Carbonatic | 28.3 | 1.4 | 837 | 107 | 41 | 391.75 | 20.73 |
| TAfi2 | 328952.5 | 4651009.6 | 0 | absence | Carbonatic | 28.7 | 1.6 | 807 | 103 | 39 | 334.09 | 11.68 |
| Tafoc | 329748.5 | 4657736.5 | 0 | absence | Carbonatic | 27.7 | 1.2 | 776 | 96 | 43 | 478.27 | 33 |
| TAlic1 | 326928.4 | 4662381.2 | 0 | absence | Carbonatic | 26.5 | 0.7 | 732 | 92 | 45 | 507.26 | 17 |
| TAlic2 | 326634.1 | 4659084 | 1 | low-medium | Carbonatic | 27.8 | 1.4 | 778 | 96 | 40 | 397.41 | 21 |
| TAlic3 | 327631.5 | 4654292.3 | 0 | absence | Carbonatic | 29 | 1.7 | 818 | 106 | 37 | 340.79 | 37 |
| Taos1 | 309028.6 | 4639924.7 | 0 | absence | Volcanic | 32 | 2.9 | 871 | 123 | 22 | 48.26 | 30.85 |
| Taos2 | 307209 | 4643727.4 | 0 | absence | Volcanic | 32 | 2.9 | 835 | 116 | 20 | 30 | 42 |
| Tapan | 315561.2 | 4641205.4 | 0 | absence | Volcanic | 31.5 | 2.8 | 872 | 123 | 26 | 107.91 | 27.44 |
| Tapbo | 310659.6 | 4639106.1 | 0 | absence | Volcanic | 31.9 | 2.9 | 885 | 126 | 23 | 48.98 | 31.34 |
| Taprl | 302101 | 4646420.1 | 0 | absence | Post-orogenic | 31.8 | 3 | 774 | 107 | 18 | 24.75 | 38 |
| Taps1 | 315235.1 | 4640303 | 0 | absence | Volcanic | 31.7 | 2.8 | 881 | 124 | 25 | 126.13 | 27.44 |
| Taps2 | 314230.1 | 4643015.3 | 0 | absence | Volcanic | 31.9 | 2.9 | 884 | 124 | 24 | 58.35 | 29.63 |
| TAsi1 | 353171.1 | 4643237.2 | 1 | high | Carbonatic | 24.3 | -0.7 | 846 | 105 | 50 | 762.78 | 4.25 |
| TAsi2 | 352758.8 | 4642538.4 | 1 | high | Carbonatic | 25.2 | -0.4 | 886 | 111 | 55 | 717.51 | 5.26 |
| Tasv1 | 317213.5 | 4641844.7 | 1 | low-medium | Volcanic | 31.3 | 2.8 | 865 | 122 | 26 | 132.25 | 22.44 |
| Tasv2 | 316585 | 4642371.4 | 1 | low-medium | Volcanic | 31.3 | 2.8 | 865 | 122 | 26 | 99.73 | 23.68 |
| TAsv3 | 314891.7 | 4643541.7 | 1 | low-medium | Volcanic | 31.7 | 2.9 | 881 | 124 | 25 | 72.93 | 29.63 |
| Tasva | 318974.5 | 4641264 | 0 | absence | Volcanic | 30.9 | 2.7 | 871 | 122 | 28 | 168.97 | 23.32 |
| Tavil | 314313.7 | 4649843.1 | 0 | absence | Carbonatic | 32.1 | 2.9 | 869 | 120 | 25 | 74.27 | 30.63 |
| Tavo1 | 266580.1 | 4654745.6 | 0 | absence | Volcanic | 29.9 | 4.1 | 324 | 43 | 5 | 97.05 | 25 |
| Tcar1 | 312839.6 | 4668432.3 | 0 | absence | Post-orogenic | 30.9 | 2.5 | 698 | 92 | 25 | 110.89 | 21.64 |
| Tcar2 | 311374.9 | 4668811.9 | 0 | absence | Post-orogenic | 31 | 2.4 | 680 | 90 | 24 | 95.06 | 25.01 |
| Tcar3 | 309219.3 | 4669051.2 | 0 | absence | Post-orogenic | 31.2 | 2.4 | 670 | 89 | 22 | 52.35 | 25.07 |
| Tcasi | 297940.5 | 4661729 | 0 | absence | Post-orogenic | 31.3 | 2.9 | 624 | 84 | 15 | 23.5 | 40 |
| Tcast | 299508.3 | 4625396.2 | 0 | absence | Volcanic | 30.3 | 3.4 | 817 | 117 | 18 | 139.42 | 37.85 |
| Tcor1 | 316505 | 4671080.9 | 0 | absence | Post-orogenic | 30.1 | 2.1 | 680 | 86 | 29 | 214.8 | 26 |
| Tcor2 | 315676.6 | 4671269.3 | 0 | absence | Post-orogenic | 30.1 | 2.1 | 668 | 85 | 29 | 202.53 | 27 |
| Tcor3 | 313728.8 | 4671060.9 | 0 | absence | Post-orogenic | 30.5 | 2.4 | 674 | 87 | 26 | 151.36 | 28 |
| Tcor4 | 312805.2 | 4670775.8 | 0 | absence | Post-orogenic | 30.6 | 2.4 | 668 | 87 | 25 | 144.44 | 25.01 |
| Tcor5 | 312037.1 | 4670741 | 1 | low-medium | Post-orogenic | 30.7 | 2.4 | 669 | 87 | 25 | 145.24 | 25.01 |
| Tcor6 | 305549.1 | 4669442.1 | 0 | absence | Post-orogenic | 31.8 | 2.5 | 668 | 89 | 19 | 26.89 | 38 |
| Tcorn | 294613.2 | 4631945.2 | 0 | absence | Volcanic | 30.5 | 3.3 | 783 | 110 | 16 | 53.4 | 38.07 |
| Tcre1 | 284317.7 | 4663585 | 0 | absence | Volcanic | 29.9 | 3 | 301 | 40 | 8 | 172.16 | 32 |
| Tcre2 | 283690.5 | 4657058.6 | 0 | absence | Volcanic | 30.5 | 3.2 | 399 | 54 | 8 | 105.55 | 34 |
| Tcre3 | 285565.5 | 4654457 | 0 | absence | Volcanic | 30.6 | 3.1 | 477 | 64 | 10 | 72.47 | 30.35 |
| Tcre4 | 286407.4 | 4653689.2 | 0 | absence | Volcanic | 30.6 | 3.1 | 482 | 65 | 10 | 41.19 | 31.74 |
| Tcre5 | 291476.9 | 4651477.2 | 0 | absence | Volcanic | 30.9 | 3.1 | 613 | 83 | 13 | 24.25 | 47.79 |
| Tcrs1 | 287622.3 | 4651918.4 | 0 | absence | Volcanic | 30.6 | 3.1 | 520 | 69 | 11 | 63.03 | 32.8 |
| Tcrs2 | 289561.8 | 4649920.2 | 0 | absence | Volcanic | 30.8 | 3.2 | 608 | 82 | 12 | 35.32 | 38.8 |
| TdxAa | 293538 | 4638131.7 | 0 | absence | Volcanic | 30.4 | 3.6 | 747 | 104 | 15 | 17.32 | 34.66 |
| TdxAb | 293115.7 | 4638129.1 | 0 | absence | Volcanic | 30.4 | 3.6 | 747 | 104 | 15 | 16.23 | 34.66 |
| Tev1 | 302892.7 | 4674532.9 | 0 | absence | Post-orogenic | 31.3 | 2.9 | 620 | 81 | 18 | 16.58 | 35.73 |
| Tev10 | 279241.8 | 4631510.1 | 0 | absence | Post-orogenic | 30 | 4.1 | 728 | 95 | 12 | 4.96 | 38 |
| Tev11 | 273850.7 | 4628651.6 | 0 | absence | Post-orogenic | 29.6 | 4.6 | 728 | 95 | 11 | 3.74 | 38 |
| Tev12 | 303034.2 | 4674324.4 | 0 | absence | Post-orogenic | 31.4 | 2.8 | 622 | 82 | 18 | 16.68 | 35.73 |
| Tev13 | 302921.1 | 4675126.1 | 0 | absence | Post-orogenic | 31.3 | 2.9 | 620 | 81 | 18 | 22.98 | 34.34 |
| Tev14 | 302296.8 | 4676157 | 0 | absence | Post-orogenic | 30.4 | 2.7 | 518 | 67 | 18 | 24.45 | 18.53 |
| Tev15 | 303660.1 | 4677247.5 | 0 | absence | Post-orogenic | 31 | 2.7 | 602 | 79 | 19 | 27.18 | 35 |
| Tev2 | 304531.1 | 4670883.1 | 0 | absence | Post-orogenic | 31.9 | 2.6 | 651 | 86 | 19 | 11.37 | 31.79 |
| Tev3 | 304775.9 | 4667803.1 | 0 | absence | Post-orogenic | 31.8 | 2.5 | 672 | 90 | 19 | 12.93 | 38 |
| Tev4 | 301642.3 | 4662191.8 | 0 | absence | Post-orogenic | 31.7 | 2.8 | 660 | 89 | 17 | 4.81 | 41 |
| Tev5 | 294209 | 4653942.4 | 0 | absence | Post-orogenic | 31.1 | 3.1 | 636 | 87 | 14 | 5.85 | 38 |
| Tev6 | 292851.4 | 4651338.1 | 0 | absence | Post-orogenic | 31 | 3.2 | 635 | 86 | 14 | 7.56 | 50 |
| Tev7 | 290745.6 | 4645804.6 | 0 | absence | Post-orogenic | 30.4 | 3.6 | 656 | 89 | 14 | 23.91 | 38 |
| Tev8 | 285824.4 | 4633911.5 | 0 | absence | Post-orogenic | 29.9 | 3.9 | 709 | 96 | 12 | 8.21 | 47.79 |
| Tev9 | 285383.9 | 4631068.4 | 0 | absence | Post-orogenic | 29.8 | 3.9 | 733 | 100 | 13 | 10.63 | 42 |
| Tfar3b | 310646.4 | 4678279.8 | 1 | low-medium | Post-orogenic | 30.8 | 2.4 | 644 | 83 | 23 | 99.51 | 21.66 |
| Tfar5 | 304613 | 4675512.2 | 0 | absence | Post-orogenic | 31.2 | 2.5 | 604 | 79 | 20 | 40.61 | 32.52 |
| Tfar6 | 304952.4 | 4675703.9 | 0 | absence | Post-orogenic | 31.2 | 2.5 | 604 | 79 | 20 | 60.07 | 31.42 |
| Tfior | 309415.9 | 4661603.7 | 0 | absence | Post-orogenic | 31.2 | 2.5 | 708 | 96 | 23 | 72.93 | 22.32 |
| Tgal1 | 279450.7 | 4649542.8 | 0 | absence | Volcanic | 30.3 | 3.5 | 500 | 66 | 9 | 73.52 | 24.4 |
| Tgal2 | 279092.7 | 4633413.9 | 0 | absence | Post-orogenic | 29.9 | 4.1 | 707 | 93 | 12 | 3.36 | 43.79 |
| Tlepr | 298625.3 | 4665747.4 | 0 | absence | Volcanic | 31.2 | 2.8 | 569 | 76 | 15 | 47.5 | 36.22 |
| Tmal1 | 288474.7 | 4624583.9 | 0 | absence | Volcanic | 29.4 | 3.9 | 778 | 108 | 14 | 23.18 | 30.65 |
| Tmal2 | 283992.9 | 4629452.3 | 0 | absence | Post-orogenic | 29.6 | 4 | 731 | 99 | 13 | 14.32 | 43.79 |
| Tmar1 | 289617.5 | 4655932.5 | 0 | absence | Volcanic | 30.9 | 3 | 537 | 72 | 12 | 61.25 | 42.22 |
| Tmar2 | 290829.1 | 4653833.8 | 0 | absence | Volcanic | 31 | 3 | 575 | 78 | 12 | 25 | 33.39 |
| Tmos1 | 310013.3 | 4665480.3 | 0 | absence | Post-orogenic | 31.4 | 2.4 | 707 | 94 | 23 | 94.88 | 19.47 |
| Tmos2 | 308820.5 | 4666724.6 | 0 | absence | Post-orogenic | 31.4 | 2.4 | 684 | 91 | 22 | 85.93 | 20.07 |
| Tmos3 | 306969 | 4667033.7 | 0 | absence | Post-orogenic | 31.7 | 2.5 | 688 | 93 | 20 | 40.57 | 39.79 |
| TOR1 | 348928 | 4678423.9 | 0 | absence | Clay flysch | 26.3 | -0.6 | 853 | 107 | 43 | 616.68 | 24 |
| Trej1a | 283611.6 | 4673633 | 0 | absence | Volcanic | 30.5 | 2.5 | 285 | 37 | 8 | 165.29 | 18.3 |
| Trej1b | 283619.1 | 4673665.3 | 0 | absence | Volcanic | 30.5 | 2.5 | 285 | 37 | 8 | 162.95 | 18.3 |
| Trej2 | 286713.9 | 4676530.8 | 0 | absence | Volcanic | 30.3 | 2.5 | 368 | 47 | 10 | 109.9 | 20.27 |
| TrioM1 | 297061.6 | 4671858.4 | 0 | absence | Volcanic | 30.8 | 2.7 | 509 | 67 | 15 | 111.51 | 21.66 |
| TrioM2 | 297619.2 | 4668980.7 | 0 | absence | Volcanic | 31.1 | 2.7 | 542 | 72 | 16 | 84.98 | 30.04 |
| TrioM3 | 301030.6 | 4666168.4 | 0 | absence | Volcanic | 31.8 | 2.7 | 638 | 85 | 17 | 33.25 | 30 |
| TRON | 357888.6 | 4732626.8 | 1 | high | Arenaceous flysch | 24.4 | -1.1 | 849 | 97 | 42 | 723.26 | 21 |
| Ttmol | 289221.4 | 4674742.6 | 0 | absence | Volcanic | 30.4 | 2.4 | 358 | 46 | 11 | 167 | 22.63 |
| Ttoc | 292788.6 | 4636147.2 | 0 | absence | Volcanic | 30.3 | 3.6 | 756 | 105 | 15 | 26.45 | 33.09 |
| TtoM1 | 292676.5 | 4635946.2 | 0 | absence | Volcanic | 30.3 | 3.6 | 756 | 105 | 15 | 21.64 | 33.09 |
| TtoM2 | 292382 | 4636325.2 | 0 | absence | Volcanic | 30.3 | 3.6 | 756 | 105 | 15 | 13.57 | 34.8 |
| Ttor1 | 292064.7 | 4664071.9 | 0 | absence | Volcanic | 30.3 | 2.9 | 438 | 58 | 12 | 140.41 | 43.04 |
| Ttor2 | 291583.2 | 4657560 | 0 | absence | Volcanic | 30.9 | 3 | 546 | 74 | 12 | 46.69 | 40.8 |
| Ttrav | 288401.1 | 4648190.1 | 0 | absence | Post-orogenic | 30.3 | 3.4 | 571 | 77 | 12 | 39.87 | 38.8 |
| TUR1 | 335942.1 | 4667093.9 | 1 | low-medium | Carbonatic | 26.7 | 0.1 | 785 | 99 | 47 | 549.73 | 6.3 |
| TUR3 | 327636.2 | 4685488.8 | 1 | low-medium | Post-orogenic | 27.7 | 0.8 | 701 | 87 | 43 | 420.53 | 5.42 |
| TUR4 | 326057.2 | 4690781.8 | 1 | high | Post-orogenic | 27.8 | 0.8 | 686 | 85 | 42 | 398.74 | 8.53 |
| Turbi | 253658.3 | 4653714 | 0 | absence | Post-orogenic | 29.8 | 4.7 | 530 | 70 | 7 | 8.52 | 43.28 |
| Tvall | 290905.7 | 4629286.4 | 0 | absence | Volcanic | 30 | 3.5 | 767 | 107 | 14 | 35.5 | 38.11 |
| Tvalu | 294367.5 | 4655318.3 | 0 | absence | Post-orogenic | 31.2 | 3.1 | 628 | 86 | 14 | 19.91 | 37 |
| Vacc1 | 262055.3 | 4655884.2 | 0 | absence | Volcanic | 29.3 | 4.4 | 292 | 39 | 5 | 68.19 | 21.63 |
| Vacc2 | 259949.2 | 4652093.4 | 0 | absence | Post-orogenic | 30 | 4.6 | 496 | 65 | 7 | 24.89 | 30.86 |
| VAR1 | 338441.1 | 4680351.8 | 1 | high | Arenaceous flysch | 24.9 | -0.2 | 762 | 96 | 39 | 546.35 | 4.53 |
| VE01 | 349643 | 4716686.6 | 1 | high | Arenaceous flysch | 24.6 | -1.1 | 829 | 98 | 39 | 799.34 | 8 |
| VE02 | 343946.2 | 4710083.4 | 1 | high | Carbonatic | 25.6 | -1.1 | 806 | 97 | 38 | 717.62 | 11 |
| VE03 | 345412.1 | 4709530.1 | 1 | high | Carbonatic | 25.1 | -1.1 | 810 | 97 | 38 | 724.67 | 9.63 |
| VE04 | 341739.9 | 4698727.4 | 1 | high | Carbonatic | 25.4 | -0.5 | 799 | 98 | 39 | 493.96 | 35 |
| VE05 | 338442.5 | 4695447.4 | 1 | high | Carbonatic | 27.1 | 0.2 | 780 | 96 | 44 | 439.02 | 20 |
| VE06 | 334477.8 | 4692398.2 | 0 | absence | Carbonatic | 27.8 | 0.5 | 759 | 93 | 46 | 404.21 | 31 |
| VE07 | 328230.2 | 4695662.3 | 0 | absence | Post-orogenic | 28.5 | 0.7 | 704 | 85 | 43 | 389.92 | 36.73 |
| VE08 | 324830.3 | 4696674 | 0 | absence | Post-orogenic | 28.9 | 0.7 | 679 | 82 | 42 | 385.58 | 43 |
| VE09 | 320598.4 | 4699794.2 | 1 | low-medium | Post-orogenic | 28.9 | 0.6 | 646 | 78 | 38 | 372.87 | 30 |
| VE10 | 318348.8 | 4704008.6 | 1 | low-medium | Post-orogenic | 28.4 | 0.8 | 625 | 75 | 36 | 368.82 | 17.63 |
| VE11 | 318790 | 4706385.3 | 0 | absence | Post-orogenic | 28 | 0.8 | 627 | 75 | 38 | 368.65 | 18.34 |
| VE12 | 314674.8 | 4708217.3 | 0 | absence | Post-orogenic | 27.9 | 1.1 | 601 | 72 | 34 | 366.48 | 9.37 |
| VE13 | 322313 | 4706166.4 | 1 | low-medium | Carbonatic | 28.5 | 0.6 | 661 | 79 | 41 | 377.9 | 26 |
| VE14 | 317821.3 | 4706949.7 | 1 | low-medium | Post-orogenic | 28 | 0.8 | 627 | 75 | 38 | 376.35 | 25.85 |
| VT001 | 238738.7 | 4743034.8 | 0 | absence | Post-orogenic | 28.8 | 1.8 | 517 | 64 | 17 | 266.85 | 8 |
| VT002 | 242444.2 | 4739419 | 0 | absence | Post-orogenic | 28.7 | 1.8 | 495 | 62 | 17 | 246.76 | 23 |
| VT003 | 249081.8 | 4740419.3 | 0 | absence | Post-orogenic | 28 | 2 | 497 | 61 | 17 | 217.01 | 5.29 |
| VT004 | 251321.8 | 4739785 | 0 | absence | Clay flysch | 28.2 | 1.9 | 493 | 61 | 17 | 196.76 | 5.26 |
| VT005 | 245941.9 | 4738571 | 0 | absence | Post-orogenic | 28.4 | 1.9 | 492 | 61 | 17 | 235.76 | 5.85 |
| VT006 | 248190.6 | 4736157 | 0 | absence | Clay flysch | 27.7 | 1.4 | 468 | 58 | 17 | 349.42 | 5.44 |
| VT007 | 245413.1 | 4739100.9 | 0 | absence | Clay flysch | 27.9 | 1.8 | 495 | 61 | 18 | 282.39 | 17.73 |
| VT008 | 244839.4 | 4735119.2 | 0 | absence | Volcanic | 27.8 | 1.5 | 468 | 57 | 18 | 439.42 | 25 |
| VT009 | 243683.5 | 4736191.5 | 0 | absence | Volcanic | 27.9 | 1.5 | 473 | 58 | 18 | 389.8 | 23 |
| VT010 | 242770.4 | 4741896.8 | 0 | absence | Clay flysch | 27.7 | 1.8 | 503 | 62 | 18 | 342.49 | 14.73 |
| VT011 | 242420 | 4740044.5 | 0 | absence | Post-orogenic | 28.7 | 1.9 | 499 | 63 | 17 | 258.52 | 23 |
| VT012 | 239972.2 | 4737879.1 | 1 | high | Clay flysch | 28 | 1.5 | 484 | 59 | 18 | 321.85 | 18.34 |
| VT013 | 239597.4 | 4745927.2 | 0 | absence | Post-orogenic | 28.7 | 1.8 | 520 | 65 | 18 | 309.16 | 19 |
| VT014 | 239199.8 | 4743318.7 | 0 | absence | Post-orogenic | 28.8 | 1.8 | 513 | 64 | 17 | 273.1 | 19 |
| VT015 | 236082.8 | 4741977.1 | 0 | absence | Clay flysch | 28.4 | 1.8 | 511 | 64 | 17 | 324.97 | 11.4 |
| VT016 | 237849.2 | 4744803.6 | 0 | absence | Post-orogenic | 28.9 | 1.8 | 519 | 65 | 17 | 278.66 | 19 |
| VT017 | 238931.9 | 4736498 | 0 | absence | Volcanic | 27.7 | 1.5 | 480 | 59 | 18 | 397 | 4.68 |
| VT018 | 237283.4 | 4719148.7 | 0 | absence | Volcanic | 28.3 | 2.1 | 414 | 51 | 13 | 396.28 | 13.34 |
| VT019 | 233213.3 | 4719319.4 | 0 | absence | Volcanic | 28.1 | 2.3 | 426 | 53 | 13 | 354.18 | 12.42 |
| VT020 | 232966 | 4718689.7 | 1 | low-medium | Volcanic | 28 | 2.3 | 424 | 53 | 13 | 342.36 | 13.37 |
| VT021 | 234053.3 | 4718094.9 | 1 | low-medium | Volcanic | 28.4 | 2.3 | 422 | 53 | 12 | 349.04 | 12.53 |
| VT022 | 229350.8 | 4716653.2 | 0 | absence | Volcanic | 28.5 | 2.8 | 447 | 57 | 12 | 224.56 | 11.26 |
| VT023 | 227776.4 | 4714737.1 | 0 | absence | Volcanic | 29.1 | 2.7 | 448 | 57 | 11 | 241.13 | 11.25 |
| VT024 | 225171.3 | 4714396.7 | 0 | absence | Volcanic | 29.3 | 2.8 | 468 | 61 | 11 | 149.55 | 11.26 |
| VT025 | 221706.9 | 4713447.2 | 0 | absence | Volcanic | 28.8 | 3.1 | 496 | 65 | 11 | 100.26 | 11.25 |
| VT026 | 221949.6 | 4716217.5 | 0 | absence | Volcanic | 29.1 | 2.8 | 478 | 62 | 11 | 148.35 | 11.32 |
| VT027 | 219296 | 4717293.3 | 0 | absence | Volcanic | 29.4 | 2.9 | 505 | 66 | 12 | 110.18 | 7.42 |
| VT028 | 221271.8 | 4713135.6 | 0 | absence | Clay flysch | 28.8 | 3.1 | 496 | 65 | 11 | 95.46 | 7.25 |
| VT029 | 226583.4 | 4711047.5 | 0 | absence | Volcanic | 29.3 | 2.7 | 452 | 58 | 11 | 222.73 | 12.27 |
| VT030 | 221865.1 | 4712216.6 | 0 | absence | Clay flysch | 28.9 | 3.1 | 493 | 64 | 11 | 90.09 | 7.25 |
| VT031 | 222973.3 | 4710550.6 | 0 | absence | Volcanic | 29.2 | 3.2 | 487 | 63 | 11 | 130.38 | 7.25 |
| VT032 | 223309.7 | 4702192.3 | 0 | absence | Post-orogenic | 30.5 | 3.6 | 511 | 68 | 10 | 37.17 | 13.34 |
| VT033 | 223594.3 | 4698734.7 | 0 | absence | Post-orogenic | 30.7 | 3.7 | 520 | 69 | 9 | 22.88 | 16 |
| VT034 | 227621.2 | 4700231.9 | 0 | absence | Volcanic | 30.5 | 3.5 | 497 | 65 | 9 | 67.03 | 20 |
| VT035 | 231666.3 | 4707621.8 | 0 | absence | Volcanic | 29.4 | 2.9 | 434 | 56 | 10 | 198.1 | 23 |
| VT036 | 226080.9 | 4697280.8 | 0 | absence | Volcanic | 30.5 | 3.6 | 503 | 66 | 9 | 47.6 | 20 |
| VT037 | 220935.9 | 4695087.3 | 0 | absence | Post-orogenic | 30.1 | 3.8 | 524 | 71 | 9 | 6.38 | 27.05 |
| VT038 | 218022 | 4692309.5 | 0 | absence | Post-orogenic | 29.5 | 4 | 528 | 72 | 9 | 2.01 | 15.47 |
| VT039 | 245492.6 | 4727030.8 | 0 | absence | Volcanic | 27.9 | 2.9 | 431 | 54 | 14 | 314.6 | 22 |
| VT040 | 253949.4 | 4723111.1 | 0 | absence | Volcanic | 28 | 2.4 | 395 | 50 | 13 | 329.98 | 19 |
| VT041 | 254521.1 | 4720646.3 | 1 | low-medium | Volcanic | 27.6 | 1.9 | 381 | 47 | 14 | 392.88 | 14 |
| VT042 | 245786.3 | 4706225.1 | 0 | absence | Volcanic | 30.2 | 2.6 | 377 | 49 | 9 | 230.98 | 13.53 |
| VT043 | 246226.8 | 4709378.8 | 0 | absence | Volcanic | 29.6 | 2.5 | 372 | 47 | 10 | 274.71 | 13.32 |
| VT044 | 243396.5 | 4700266.8 | 0 | absence | Volcanic | 30.3 | 3 | 399 | 52 | 9 | 132 | 27 |
| VT045 | 243117.9 | 4698675.7 | 0 | absence | Volcanic | 30.3 | 3 | 400 | 52 | 8 | 101.27 | 18.63 |
| VT046 | 245471.3 | 4697937 | 0 | absence | Volcanic | 30.3 | 2.9 | 398 | 51 | 9 | 112.32 | 13.34 |
| VT047 | 245817.7 | 4695153.3 | 0 | absence | Volcanic | 30.2 | 3 | 397 | 51 | 8 | 126.06 | 5.31 |
| VT048 | 243826 | 4694310.8 | 0 | absence | Allochthonous flysch | 30.2 | 3.1 | 386 | 50 | 8 | 86.66 | 12.28 |
| VT049 | 248114.1 | 4692678.5 | 0 | absence | Volcanic | 30.2 | 2.9 | 369 | 47 | 8 | 133.76 | 6.44 |
| VT050 | 250602 | 4691688.8 | 0 | absence | Volcanic | 30.5 | 2.8 | 349 | 45 | 7 | 163.2 | 13.85 |
| VT051 | 251746 | 4685348.2 | 0 | absence | Volcanic | 30 | 3.2 | 327 | 42 | 6 | 170.98 | 14.34 |
| VT052 | 253015.1 | 4701452.3 | 0 | absence | Volcanic | 30.7 | 2.6 | 355 | 46 | 9 | 179.84 | 18 |
| VT053 | 249319.5 | 4696048.8 | 0 | absence | Volcanic | 30.3 | 2.8 | 370 | 47 | 8 | 147.64 | 13.31 |
| VT054 | 256424.3 | 4696839.6 | 0 | absence | Volcanic | 30.5 | 2.7 | 307 | 39 | 8 | 220.75 | 21.73 |
| VT055 | 250624.8 | 4694528.9 | 0 | absence | Volcanic | 30.5 | 2.8 | 368 | 47 | 8 | 132.32 | 14.31 |
| VT056 | 234224.2 | 4687112.1 | 0 | absence | Post-orogenic | 30.4 | 3.9 | 509 | 67 | 8 | 25.36 | 12.53 |
| VT057 | 231492.1 | 4683563.1 | 0 | absence | Post-orogenic | 29.9 | 4.1 | 518 | 69 | 9 | 14.19 | 40.07 |
| VT058 | 228559.8 | 4682368.5 | 0 | absence | Post-orogenic | 29.5 | 4.3 | 537 | 72 | 9 | 6.85 | 22.13 |
| VT059 | 222954.7 | 4688876.2 | 0 | absence | Post-orogenic | 29.6 | 4 | 531 | 71 | 9 | 5.2 | 9.85 |
| VT060 | 226925.8 | 4693791.2 | 0 | absence | Post-orogenic | 30.4 | 3.7 | 507 | 67 | 9 | 24.97 | 12.41 |
| VT061 | 239169.8 | 4688257.8 | 0 | absence | Allochthonous flysch | 30.1 | 3.7 | 473 | 62 | 8 | 39.81 | 12.32 |
| VT062 | 275540.8 | 4710850.2 | 0 | absence | Post-orogenic | 30.3 | 2.3 | 406 | 52 | 13 | 66.08 | 23.79 |
| VT063 | 274570.2 | 4709782.5 | 0 | absence | Post-orogenic | 30.2 | 2.2 | 388 | 50 | 12 | 70.93 | 23.52 |
| VT064 | 265919.4 | 4708179.6 | 0 | absence | Volcanic | 30.2 | 2.2 | 349 | 44 | 11 | 210.01 | 19.26 |
| VT065 | 271975.2 | 4714610.6 | 0 | absence | Volcanic | 30.2 | 2.2 | 417 | 53 | 13 | 97.02 | 26.01 |
| VT066 | 263300 | 4713052.2 | 0 | absence | Volcanic | 29.7 | 1.8 | 353 | 43 | 12 | 338.21 | 23.44 |
| VT067 | 272841.8 | 4719982.3 | 0 | absence | Post-orogenic | 30.2 | 2.3 | 441 | 56 | 14 | 82.5 | 21.8 |
| VT068 | 271589.3 | 4721545.1 | 0 | absence | Post-orogenic | 30.2 | 2.3 | 442 | 56 | 14 | 96.1 | 22.8 |
| VT069 | 271683.4 | 4723934.6 | 1 | low-medium | Post-orogenic | 30.1 | 2.2 | 448 | 56 | 15 | 120.25 | 22.91 |
| VT070 | 274762.5 | 4704834.5 | 0 | absence | Volcanic | 29.4 | 2.1 | 331 | 41 | 12 | 271.65 | 19.94 |
| VT071 | 283993.9 | 4703639.8 | 0 | absence | Post-orogenic | 29.9 | 2.2 | 390 | 49 | 14 | 146.86 | 42.79 |
| VT072 | 288046.2 | 4694213.2 | 0 | absence | Volcanic | 30.7 | 2.6 | 410 | 52 | 13 | 73.2 | 29.41 |
| VT073 | 289965.4 | 4693499.9 | 0 | absence | Post-orogenic | 30.9 | 2.5 | 441 | 56 | 14 | 51.51 | 37.79 |
| VT074 | 289903.7 | 4692652 | 0 | absence | Post-orogenic | 31.1 | 2.5 | 443 | 56 | 14 | 46.07 | 41.79 |
| VT075 | 290221.5 | 4690660.3 | 0 | absence | Post-orogenic | 31.1 | 2.5 | 438 | 56 | 14 | 69.69 | 39.79 |
| VT076 | 286481.8 | 4681605.5 | 0 | absence | Volcanic | 30.3 | 2.5 | 348 | 45 | 10 | 80.39 | 13.35 |
| VT077 | 280307.8 | 4677354.3 | 0 | absence | Volcanic | 30.2 | 2.5 | 248 | 32 | 7 | 196.42 | 19.68 |
| VT078 | 286881.4 | 4684560.2 | 0 | absence | Volcanic | 30.5 | 2.4 | 383 | 50 | 11 | 81.52 | 42.02 |
| VT079 | 290465.9 | 4686970.2 | 0 | absence | Volcanic | 31.3 | 2.4 | 439 | 56 | 14 | 40.18 | 30.79 |
| VT080 | 287192.9 | 4683571.7 | 0 | absence | Volcanic | 30.5 | 2.5 | 387 | 50 | 11 | 66.75 | 30.02 |
| VT081 | 286553.6 | 4676042.9 | 0 | absence | Volcanic | 30.3 | 2.4 | 340 | 44 | 10 | 127.42 | 20.27 |
| VT084 | 259783.7 | 4672880.3 | 0 | absence | Volcanic | 28.2 | 3.2 | 183 | 24 | 4 | 309.18 | 13.53 |
| VT085 | 253440.7 | 4678580.4 | 0 | absence | Allochthonous flysch | 29.4 | 3.5 | 298 | 39 | 6 | 149.03 | 12.32 |
| VT086 | 243639.4 | 4680025.3 | 1 | low-medium | Allochthonous flysch | 29.9 | 4 | 481 | 63 | 8 | 40.28 | 12.76 |
| VT088 | 231843.4 | 4673914.7 | 0 | absence | Post-orogenic | 29 | 4.7 | 558 | 75 | 8 | 3.3 | 21.42 |
| VT089 | 236884.6 | 4676643 | 0 | absence | Post-orogenic | 30.2 | 4.3 | 542 | 71 | 8 | 7.58 | 13.35 |
| VT090 | 247286.7 | 4738156.3 | 1 | low-medium | Post-orogenic | 28 | 1.8 | 482 | 60 | 17 | 236.94 | 6.34 |
| VT091 | 247112.1 | 4712612.6 | 0 | absence | Volcanic | 28.7 | 2.8 | 372 | 47 | 11 | 306.25 | 21.26 |
| VT092 | 237945.1 | 4704863.5 | 0 | absence | Volcanic | 29.6 | 2.9 | 413 | 53 | 10 | 211.01 | 13.68 |
| VT093 | 216037.5 | 4701572.1 | 0 | absence | Post-orogenic | 30.6 | 3.8 | 529 | 71 | 10 | 35.62 | 13.35 |
| VT094 | 212097.1 | 4698799.3 | 0 | absence | Post-orogenic | 30.4 | 3.8 | 532 | 72 | 10 | 5.1 | 28.31 |
| VT095 | 270043.8 | 4724741.8 | 1 | low-medium | Post-orogenic | 29.3 | 2 | 420 | 52 | 15 | 170.83 | 18.84 |
| VT096 | 266872 | 4721803.4 | 0 | absence | Post-orogenic | 29.5 | 2.2 | 423 | 52 | 14 | 147.55 | 15.29 |
| VT097 | 269597.5 | 4719409 | 0 | absence | Volcanic | 29.4 | 2.1 | 395 | 48 | 14 | 230.06 | 17.92 |
| VT098 | 271063.2 | 4715130.3 | 0 | absence | Volcanic | 30.2 | 2.3 | 417 | 53 | 13 | 98.48 | 26.01 |
| VT099 | 252963.1 | 4671341.4 | 0 | absence | Allochthonous flysch | 29.2 | 3.8 | 340 | 44 | 6 | 110.49 | 20 |
| VT100 | 291985.7 | 4688989.3 | 0 | absence | Post-orogenic | 31.2 | 2.6 | 484 | 62 | 15 | 25.26 | 35.63 |
| VT101 | 222931.8 | 4703036.3 | 0 | absence | Post-orogenic | 30.4 | 3.6 | 503 | 67 | 10 | 39.15 | 12.53 |

### **Table S2**

Confusion matrix with values obtained for the test dataset before (in brackets) and after (in bold) the threshold optimization. Note the reduction of the false negatives.

|  |  | ***Observed*** | |
| --- | --- | --- | --- |
|  |  | *Absence* | *Presence* |
| ***Predicted*** | *Absence* | (74) **73** | (5) **3** |
|  | *Presence* | (5) **6** | (18) **20** |
